# Supplementary material for: Effect of Enzymatic, Ultrasound, and Reflux Extraction Pretreatments on the Chemical Composition of Essential Oils
Source: Molecules. 2020 Oct 20;25(20):4818. doi: 10.3390/molecules25204818 (PMC7587977; doi:10.3390/molecules25204818)
Supplement: Supplementary file 1 [file molecules-25-04818-s001.zip › Appendix 1-molecules-942617-R2.docx]

Appendix 1 of the manuscript:

Effect of Enzymatic, Ultrasound, and Reflux Extraction Pretreatments on the Chemical Composition of Essential Oils

**Anđela Miljanović ^1^, Ana Bielen ^1,^*, Dorotea Grbin ^1^, Zvonimir Marijanović ^2^, Martina Andlar ^1^, Tonči Rezić ^1^, Sunčica Roca ^3^, Igor Jerković ^2^, Dražen Vikić-Topić ^3,4^ and Maja Dent ^1,^***

^1^ Faculty of Food Technology and Biotechnology, University of Zagreb, Pierottijeva 6, 10 000 Zagreb, Croatia, [amiljanovic@pbf.hr (A.M.)](mailto:amiljanovic@pbf.hr(A.M.)); [dorotea.polo@gmail.com](mailto:dorotea.polo@gmail.com) (D.G.); [martina.andlar@gmail.com](mailto:martina.andlar@gmail.com) (M.A.); [trezic@pbf.hr](mailto:trezic@pbf.hr); [maja.dent@pbf.unizg.hr](mailto:mfeges@pbf.hr) (T.R.)

^2^ Faculty of Chemistry and Technology, University of Split, Ruđera Boškovića 35, 21 000 Split, Croatia, [zmarijanovic@ktf-split.hr](mailto:zmarijanovic@ktf-split.hr) (Z.M.) ; [igor@ktf-split.hr](mailto:igor@ktf-split.hr) (I.J.)

^3^ NMR Centre, Ruđer Bošković Institute, Bijenička cesta 54, 10 000 Zagreb, Croatia, [sroca@irb.hr](mailto:sroca@irb.hr) (S.R.); [vikic@irb.hr](mailto:dvikic@irb.hr) (D.V.-T.)

^4^ Department of Natural and Health Sciences, Juraj Dobrila University of Pula, Zagrebačka 30, 52 100 Pula, Croatia

***** Correspondence: [abielen@pbf.hr](mailto:abielen@pbf.hr); Tel: +385 98 179 3307 (A.B.); [maja.dent@pbf.unizg.hr](mailto:mfeges@pbf.hr); Tel: +385 91 444 0555 (M.D.)

Academic Editor: Petras Rimantas Venskutonis

Received: 8 September 2020; Accepted: 19 October 2020; Published: date

Experimental details and analysis of nuclear magnetic resonance (NMR) analysis spectra

The NMR spectra were recorded on a Bruker Avance 600 spectrometer using a 5-mm broad band probehead equipped with z-gradient coils, operating at 600.130 MHz for ^1^H and 150.903 MHz for ^13^C. All spectra were measured from CDCl_3_-d with tetramethylsilane (TMS) as the internal standard at 25 °C. Individual resonances were assigned on the basis of their chemical shifts, multiplicity, signal intensities, and by using correlation signals in the spectra of 2D NMR techniques: correlation spectroscopy (^1^H-^1^H COSY), total correlation spectroscopy (^1^H-^1^H TOCSY), heteronuclear multiple quantum coherence (^1^H-^13^C HMQC) and heteronuclear multiple bond correlation (^1^H-^13^C HMBC).

One-dimensional ^1^H and ^13^C NMR spectra were recorded at Bruker AV600 by using 32K and 64K data points and spectral widths of 12019 Hz and 39370 Hz for proton and carbon (APT) experiments, respectively. Digital resolution was 0.37 Hz and 0.60 Hz per point, respectively. The number of scans was 128–256 for ^1^H and ca. 34000 for 13C APT spectra. Two‑dimensional homonuclear ^1^H-^1^H COSY and ^1^H-^1^H TOCSY spectra were recorded by using 2048 points in f2 and 512 increments in f1 dimension. Increments were obtained by 8 and 24 scans each, with 9615 Hz spectral width, and by using a relaxation delay of 1.0 and 1.5 s, respectively. The zero filling of f1 data was performed to 1024 points. The digital resolution was 4.69 and 18.75 Hz per point in f2 and f1 domains, respectively. TOCSY spectra were obtained with the mixing time of 60 ms. The inverse ^1^H-13C correlation experiments, HMQC and HMBC were recorded with 2048 points in f2 dimension and 256 increments in f1 dimension, and were subsequently zero-filled to 1024 points. For each increment, 128 (HMQC) and 256 (HMBC) scans were collected, using relaxation delay of 1.0 s. The spectral widths were 9615 Hz (f2) and 36240 Hz (f1), with the corresponding resolution of 4.69 and 141.48 Hz per point in f2 and f1 dimensions, respectively. In HMBC spectra, the additional delay of 65 ms was used for detecting the long-range C–H couplings.

The atom signals of the most terpenes observed by GC-MS analysis in more than 5% of total peak area, were also obtained in recorded NMR spectra. Their assignation was made by signal chemical shifts and multiplicity in the ^1^H and ^13^C NMR spectra, and by the cross peaks in ^1^H-^1^H COSY, ^1^H-^1^H TOCSY and ^1^H-^13^C HMBC. Due to the large spin splitting within the molecules, some atom signals came in the proton spectra as very low intensity multiplet. The signal detection in spectra was also difficult by their large overlap in the higher magnetic field due to the similarity of the chemical structures of the observed molecules. As the result of that 1,8‑cineole and α‑terpineol could not be detected with certainty. The most useful for assignation were the long-range cross peaks in the HMBC spectra. Fig. 3 shows the spectra of rosemary (Fig. 3A), sage (Fig. 3B) and bay laurel (Fig. 3C) extracts with labelled atom signals of compounds found in the mixture. In the same figure, the structures of the compounds with the numbering used in NMR spectroscopy are presented. Atom signals of the linalool were fully assigned in the proton spectra because of the cross peaks found in HMBC: H-1, H-2, H-9 to C‑3; H-5, H-8, H‑10 to C-6; H-8, H-10 to C-7. In COSY spectrum its H-1 to H-2 cross peak was observed. Recorded TOCSY revealed H-4 ‒ H-5 ‒ H-6 spin system of the linalool. The methyleugenol atom signals were revealed also by HMBC cross peaks: H-5 to C-7; H-6 to C‑2, C-4; H-7 to C-3, C-4, C-8, C-9; H-10 to C-2; H-11 to C-1. The COSY experiment revealed three vicinal couplings in molecule: H-5 to H-6; H-7 to H-8; H-8 to H-9. Atom signals of camphor were observed thanks to the deshielded C-2 atom (219.5 ppm) in the ^13^C APT spectra, and HMBC cross peaks observed between: H-3b to C-2, C-5, C-1; H-8, H-9 to C-7; H-10 to C-2, C-6, C-7. The α-terpenyl acetate signals were obtained because of the acetyl group signals: methyl H-12 at 1.97 ppm and carbonyl C-11 at 170.8 ppm. In recorded HMBC spectra cross peaks between: H-2 to C-4, C-7; H-9, H-10 to C-4, C-8, C-10, were also found. Chemical shifts and multiplicities of other α-terpenyl acetate signals (H-3a, H-3b, H‑4, H-5a, H-5b, H-6, H-7) in our spectra agree with literature data [1]. Signals of the α- and β-thujone were in recorded spectra confirmed by cross peaks in COSY and/or TOCSY spectra. For α- thujone: H-5 to H-6 endo and H-6 exo; H-6 endo to H-6 exo; H-6 endo and H-6 exo to H-7; H-4 to H-10, couplings were found. For β-thujone: H-6 endo to H-6 exo and H-6 endo and H-6 exo to H-7 were observed. Thujones signals were also confirmed by literature data [2]. All atom signals of berbenon, borneole, manool and veridiflorol were not observed in recorded NMR spectra, but each of the above molecules have some signals that confirmed their presence in the spectra of the investigating mixtures. Berbenone was assigned because of its characteristic C‑2 (170.2 ppm), H‑3/C‑3 (5.73 ppm, 121.6 ppm), C-4 (204.2 ppm) and H‑1/C‑1 (2.08 ppm, 49.4 ppm) atom signals in ^1^H and ^13^C NMR spectra. Cross peaks found in the HMBC spectra were between: H-1 to C-4; H-5 to C‑2, C‑4; H‑8 to C-2. At borneol molecule, H‑2 atom signal at 3.63 ppm was easily detected because it does not overlap with other signals. In TOCSY spectra, H-2 to H-3 cross peak was observed, as well as methyl H-10 to C-2 in HMBC spectra. At manool molecule deshielded ^1^H atom signals of H-8, H-14, H‑15a and H-15b were found. In COSY spectra H-14 to H-15 cross peak was observed. In HMBC spectra H-14 and H-15 to C-13 cross peaks were found. Other signals were found by comparison with literature data [3]. Veridiflorol was also only partially assigned, since all ^1^H atom signals fall in the range of 0.8 to 1.9 ppm where they overlap with the other molecule signals of the mixture (e.g. α-thujone and camphor). According to the signal intensity and coupling constant (6.5 Hz) the one at 0.93 ppm was attributed to veridiflorol methyl H-15 atom, and the cross peaks of H-5 to H-6 and H-4 to H-15 atoms in the COSY spectrum was detected [4]. The observed NMR spectra were compared with data published in the Biological Magnetic Resonance Data Bank database where possible [5].

References:

1. Liu, X.C.; Li, Y.P.; Li, H.Q.; Deng, Z.W.; Zhou, L.; Liu, Z.L.; Du, S.S. Identification of repellent and insecticidal constituents of the essential oil of *Artemisia rupestris* L. aerial parts against *Liposcelis bostrychophila* Badonnel. *Molecules* **2013**, *18*, 10733–10746, doi:10.3390/molecules180910733.
2. Berger, S.; Sicker, D. *Classics in Spectroscopy. Isolation and Structure Elucidation of Natural Products*; Wiley-VCH: Weinheim, Germany, 2009.
3. Manool. Available online: http://www.molbase.com/en/hnmr_596-85-0-moldata-25460.html (accessed on 14 May 2020).
4. Bombarda, I.; Raharivelomanana, P.; Ramanoelina, P.A.R.; Faure, R.; Bianchini, J-P.; Gaydou, E.M. Berger, S.; Sicker, D. Classics in Spectroscopy. Isolation and Structure Elucidation of Natural Products. Wiley-VCH, Weinheim. l. *Anal. Chim. Acta* **2009**, *447*, 113–123.
5. Biological Magnetic Resonance Data Bank. Available online: http://www.bmrb.wisc.edu/ (accessed on 10 May 2020).
