# Supplementary material for: Effect of Enzymatic, Ultrasound, and Reflux Extraction Pretreatments on the Chemical Composition of Essential Oils
Source: Molecules. 2020 Oct 20;25(20):4818. doi: 10.3390/molecules25204818 (PMC7587977; doi:10.3390/molecules25204818)
Supplement: Supplementary file 1 [file molecules-25-04818-s001.zip › Supplementary file 1-molecules-942617-R2.docx]

Supplementary file 1 of the manuscript:

Effect of Enzymatic, Ultrasound, and Reflux Extraction Pretreatments on the Chemical Composition of Essential Oils

**Anđela Miljanović ^1^, Ana Bielen ^1,^*, Dorotea Grbin ^1^, Zvonimir Marijanović ^2^, Martina Andlar ^1^, Tonči Rezić ^1^, Sunčica Roca ^3^, Igor Jerković ^2^, Dražen Vikić-Topić ^3,4^ and Maja Dent ^1,^***

^1^ Faculty of Food Technology and Biotechnology, University of Zagreb, Pierottijeva 6, 10 000 Zagreb, Croatia, [amiljanovic@pbf.hr (A.M.)](mailto:amiljanovic@pbf.hr(A.M.)); [dorotea.polo@gmail.com](mailto:dorotea.polo@gmail.com) (D.G.); [martina.andlar@gmail.com](mailto:martina.andlar@gmail.com) (M.A.); [trezic@pbf.hr](mailto:trezic@pbf.hr); [maja.dent@pbf.unizg.hr](mailto:mfeges@pbf.hr) (T.R.)

^2^ Faculty of Chemistry and Technology, University of Split, Ruđera Boškovića 35, 21 000 Split, Croatia, [zmarijanovic@ktf-split.hr](mailto:zmarijanovic@ktf-split.hr) (Z.M.) ; [igor@ktf-split.hr](mailto:igor@ktf-split.hr) (I.J.)

^3^ NMR Centre, Ruđer Bošković Institute, Bijenička cesta 54, 10 000 Zagreb, Croatia, [sroca@irb.hr](mailto:sroca@irb.hr) (S.R.); [vikic@irb.hr](mailto:dvikic@irb.hr) (D.V.-T.)

^4^ Department of Natural and Health Sciences, Juraj Dobrila University of Pula, Zagrebačka 30, 52 100 Pula, Croatia

***** Correspondence: [abielen@pbf.hr](mailto:abielen@pbf.hr); Tel: +385 98 179 3307 (A.B.); [maja.dent@pbf.unizg.hr](mailto:mfeges@pbf.hr); Tel: +385 91 444 0555 (M.D.)

Academic Editor: Petras Rimantas Venskutonis

Received: 8 September 2020; Accepted: 19 October 2020; Published: date


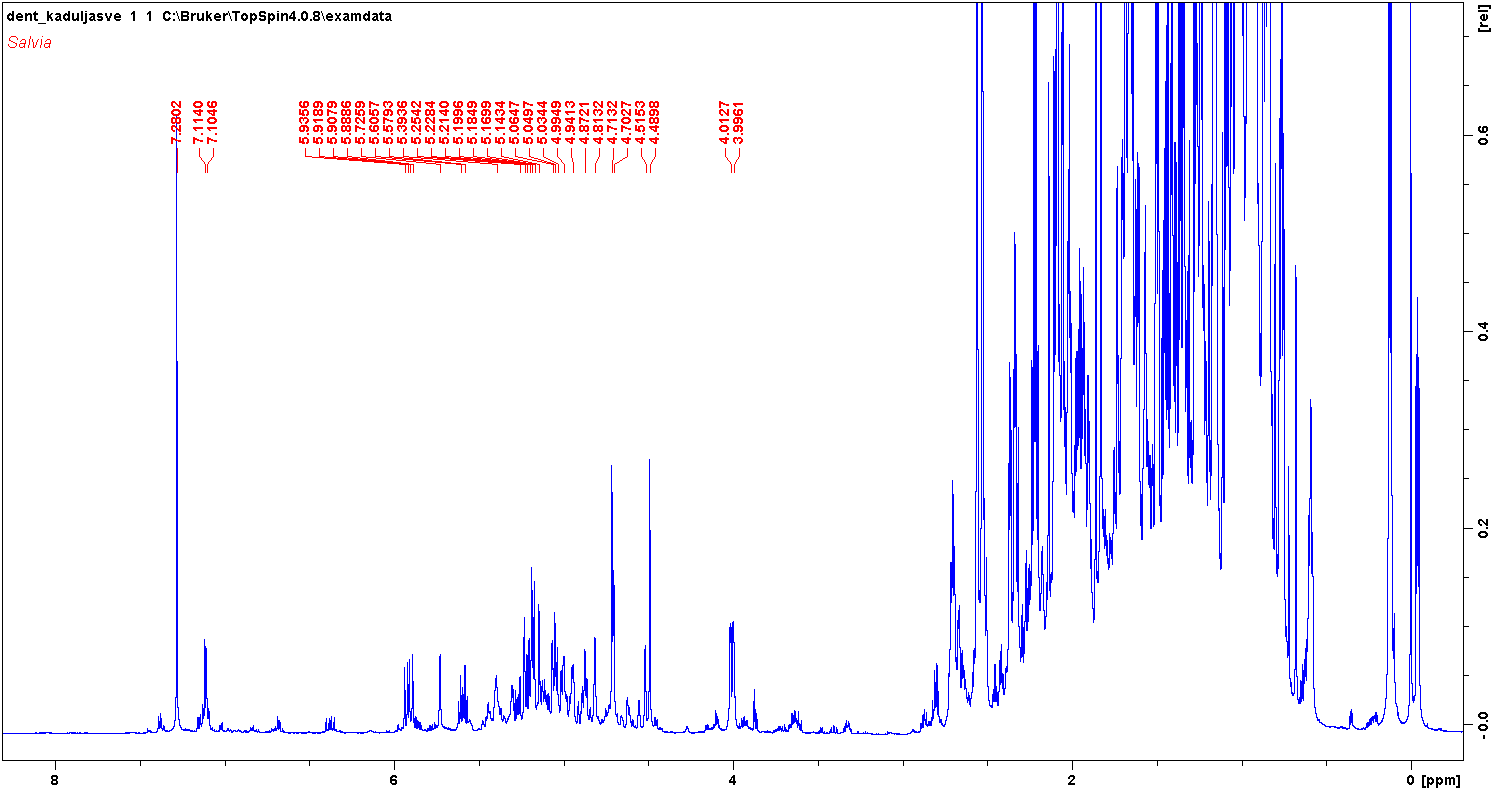

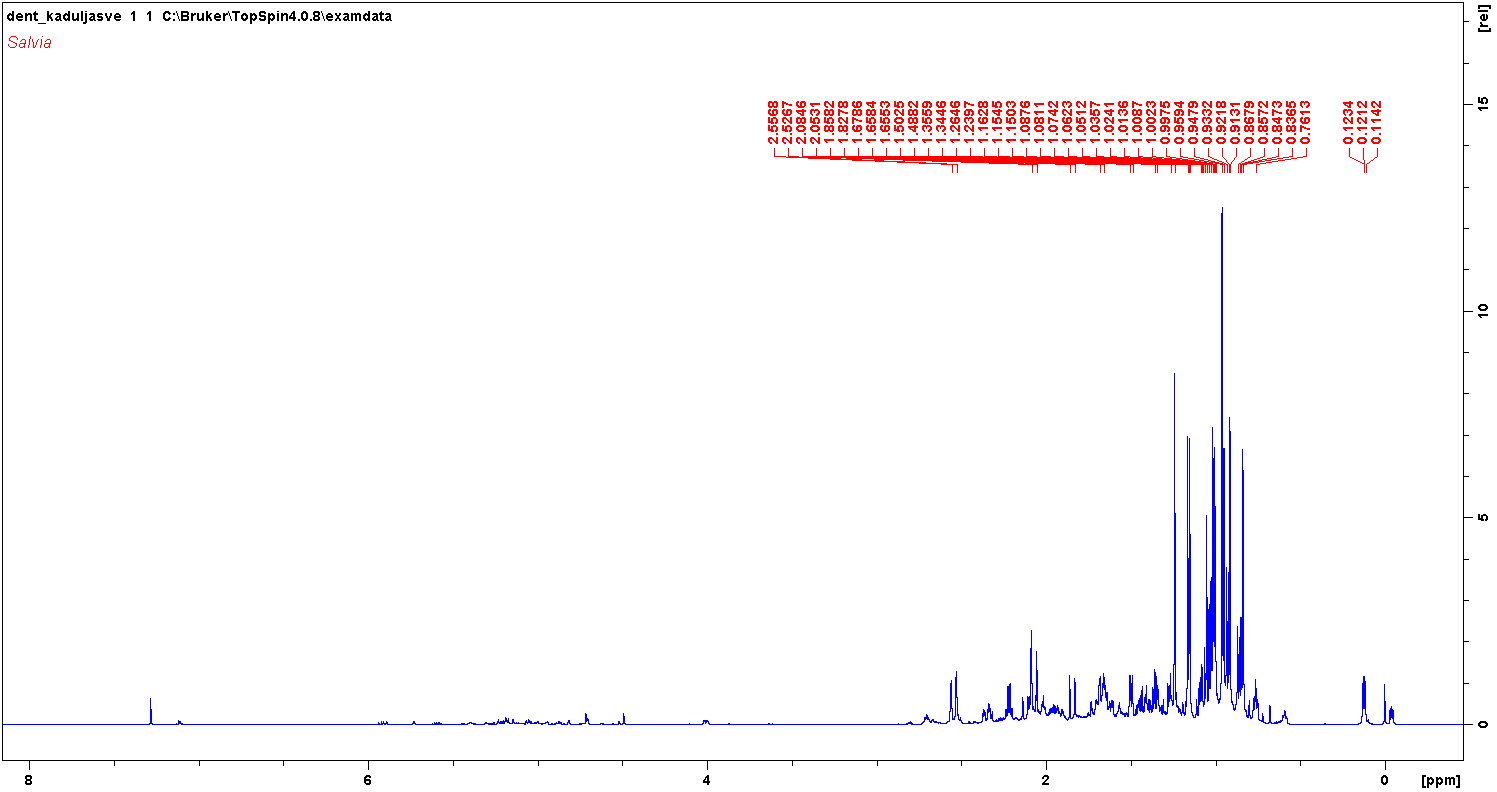


X6

**Figure 1.** Sage ^1^H NMR spectrum (600 MHz; 0.5 mL CDCl_3_; 5 mm sample tube; 25 °C; 32 *K* data points; 256 scans; 0.37 Hz/point; 1 s delay).


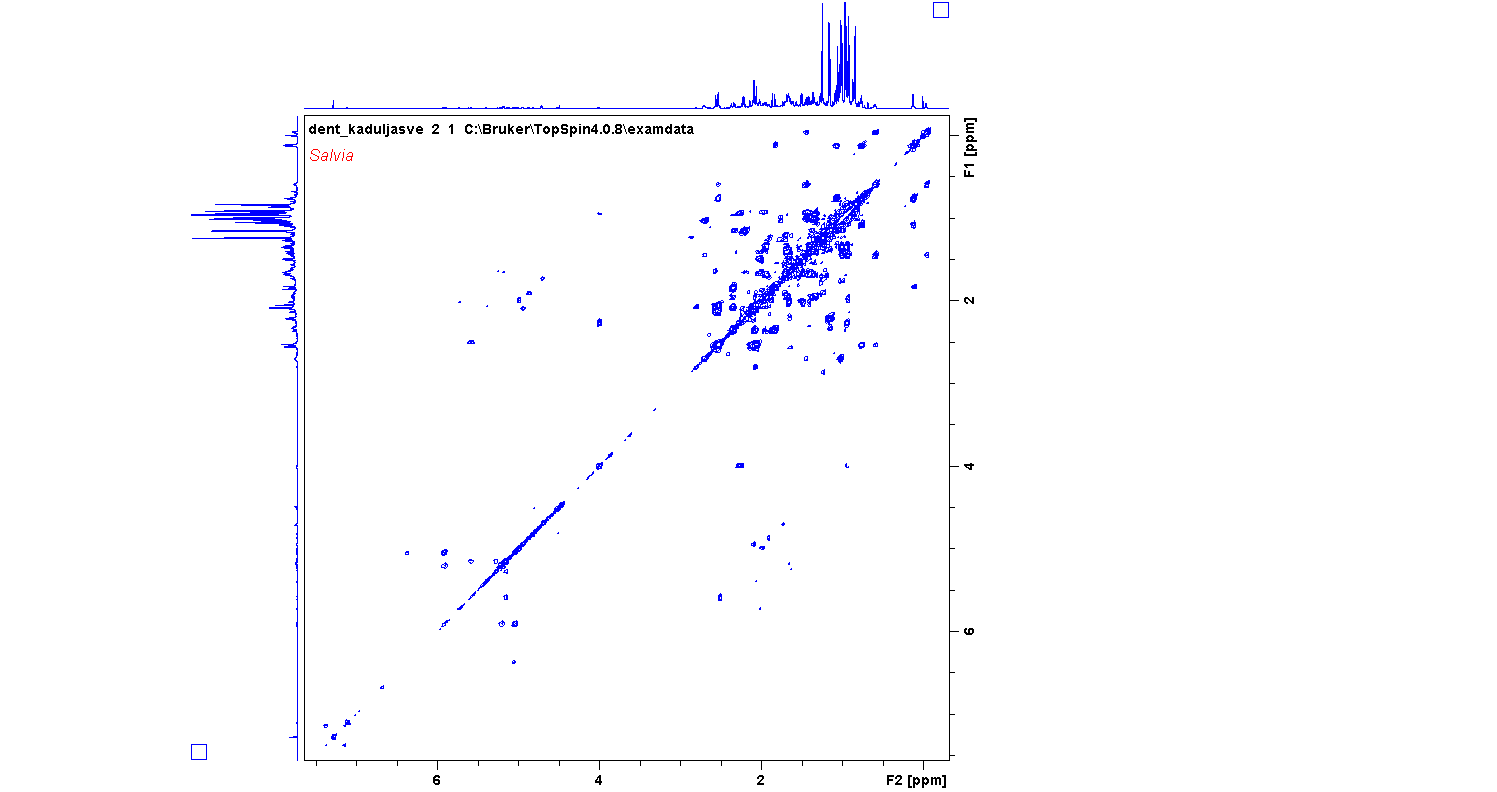


**Figure 2.** Sage ^1^H-^1^H COSY NMR spectrum (600 MHz, CDCl_3_*-d*, 25 °C).


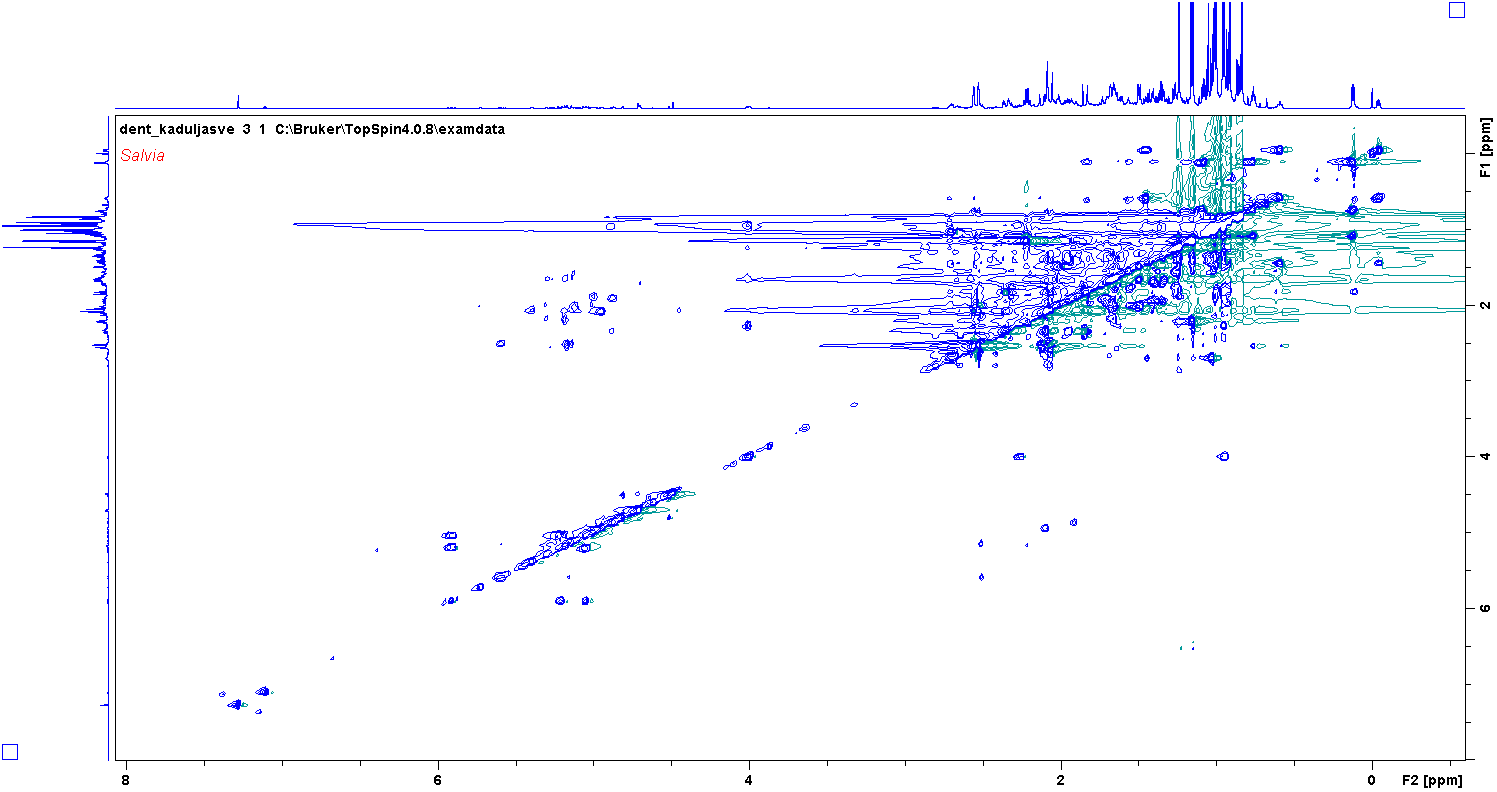


**Figure 3.** Sage ^1^H-^1^H TOCSY NMR spectrum (600 MHz, CDCl_3_-*d*, 25 °C).


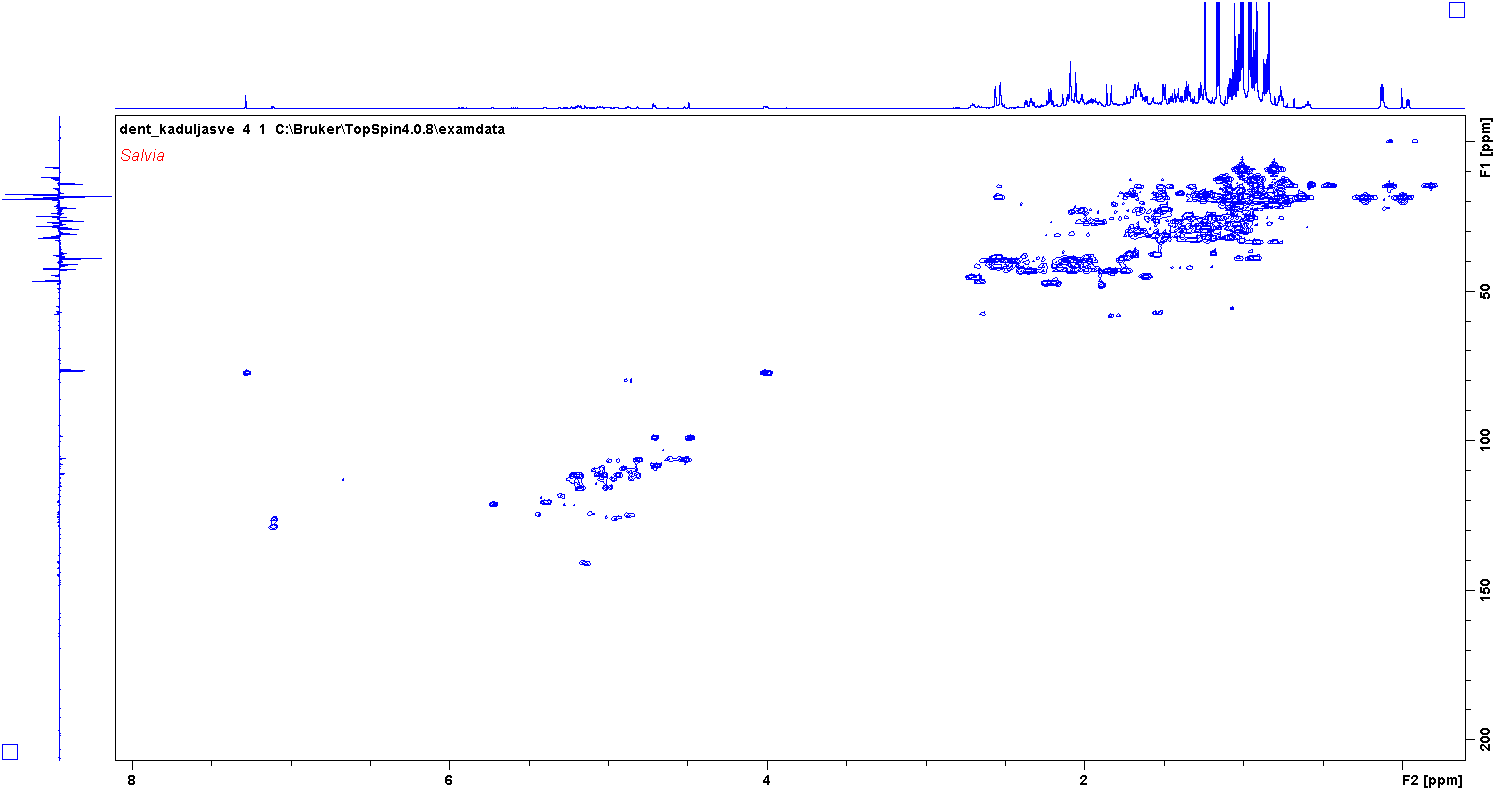


**Figure 4.** Sage ^1^H-^13^C HMQC NMR spectrum (CDCl_3_-*d*, 25 °C). The 600 MHz ^1^H NMR spectrum is shown at the top edge, and a 150 MHz ^13^C NMR spectrum at the left-hand edge.


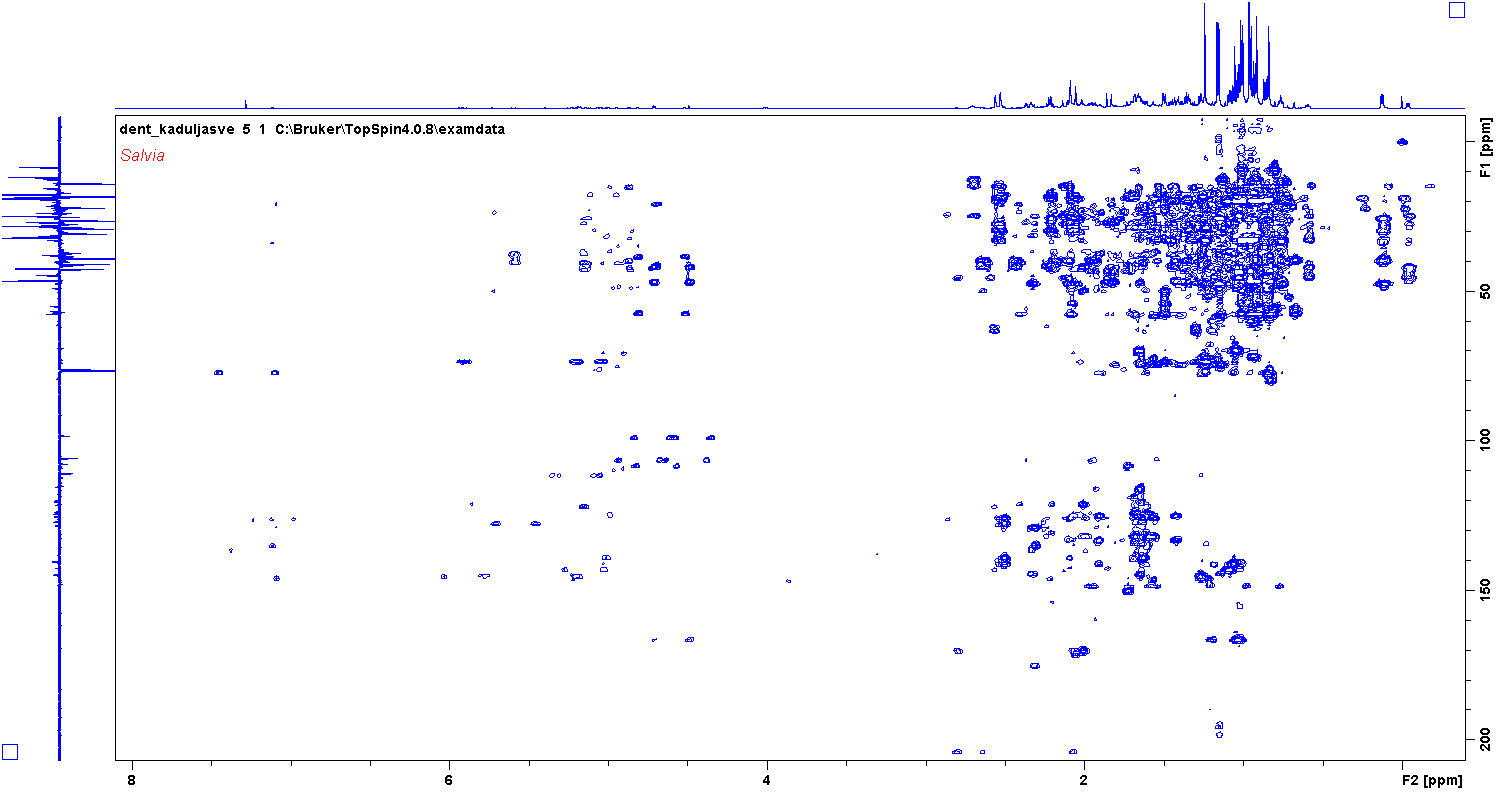


**Figure 5.** Sage ^1^H-^13^C HMBC NMR spectrum (CDCl_3_-*d*, 25 °C). The 600 MHz ^1^H NMR spectrum is shown at the top edge, and a 150 MHz ^13^C NMR spectrum at the left-hand edge.


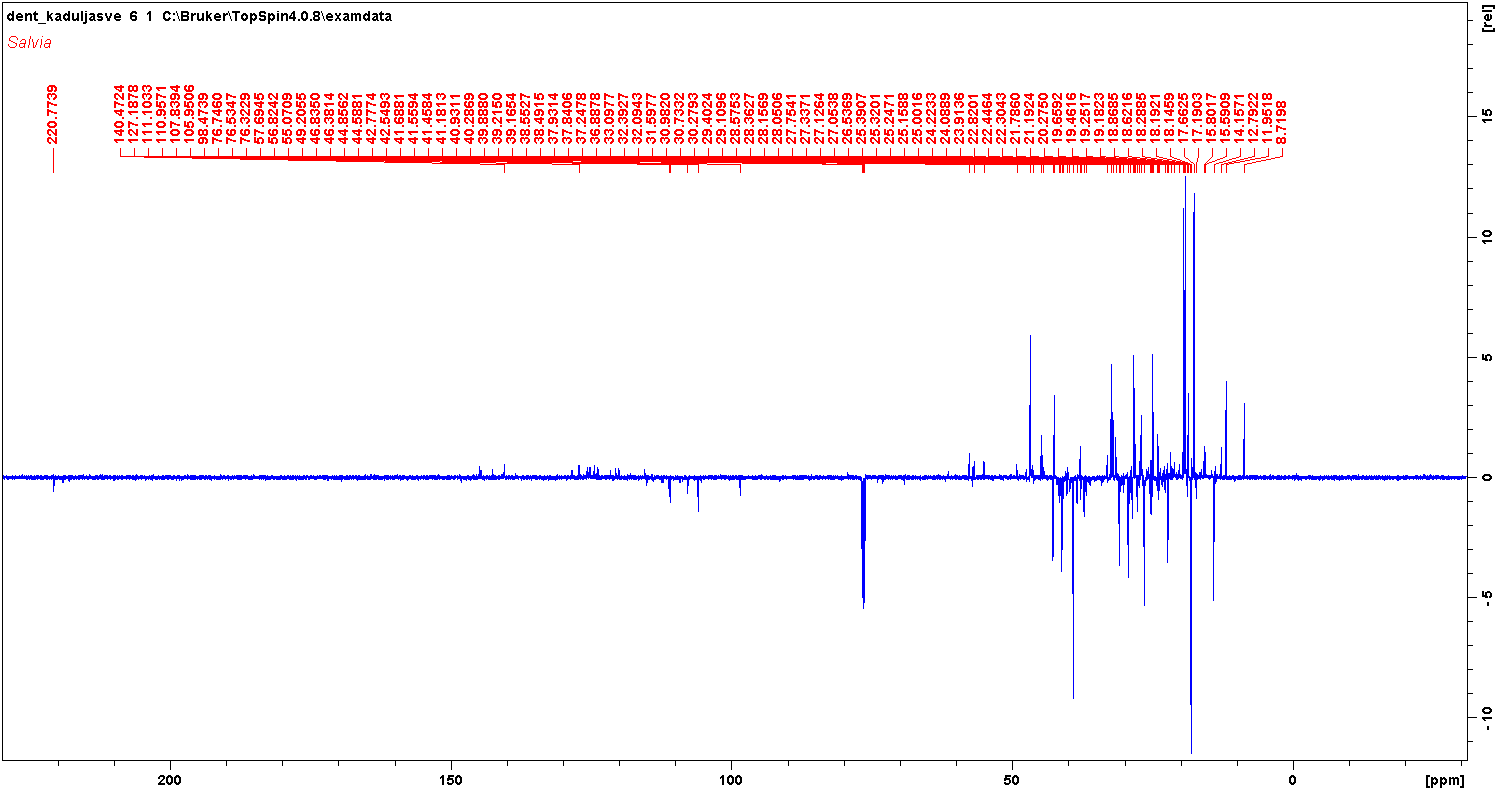


**Figure 6.** Sage ^13^C APT NMR spectrum (150 MHz, 0.5 mL CDCl_3_; 5 mm sample tube; 25 °C; 64 *K* data points; 44506 scans; 0.60 Hz/point; 1 s delay).


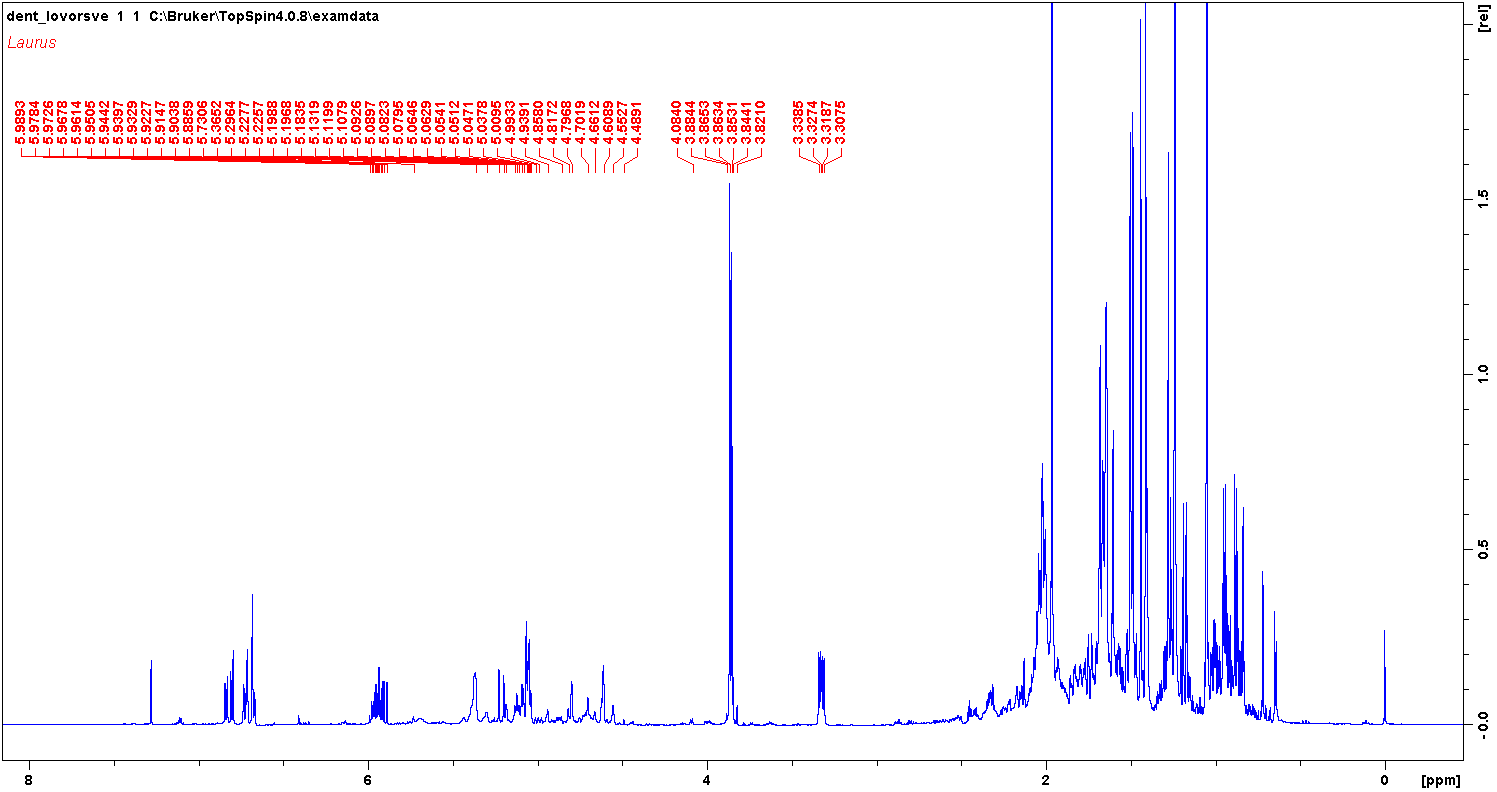

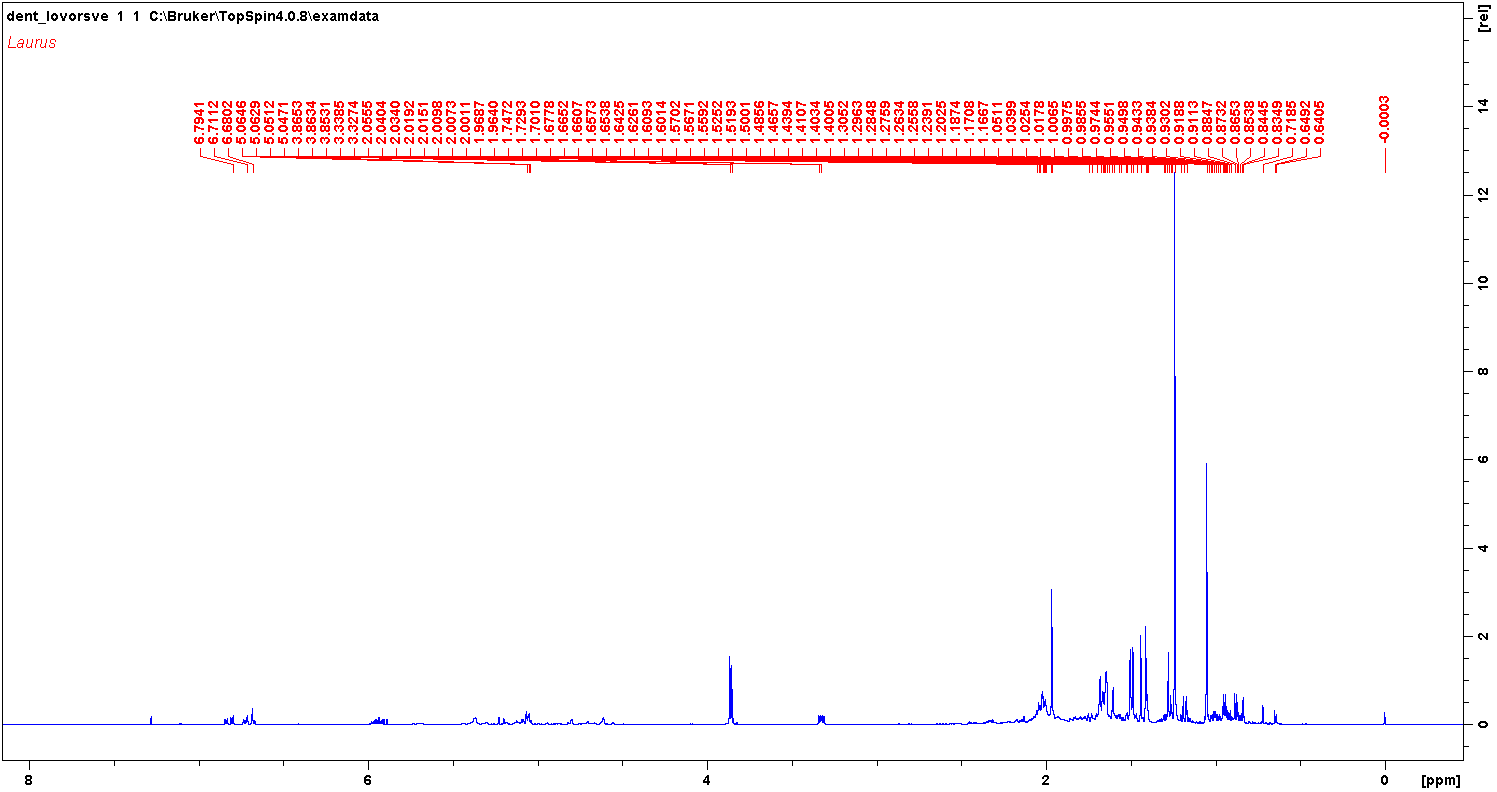


X6

**Figure 7.** Bay laurel ^1^H NMR spectrum (600 MHz, 0.5 mL CDCl_3_; 5 mm sample tube; 25 °C; 32 *K* data points; 256 scans; 0.37 Hz/point; 1 s delay).


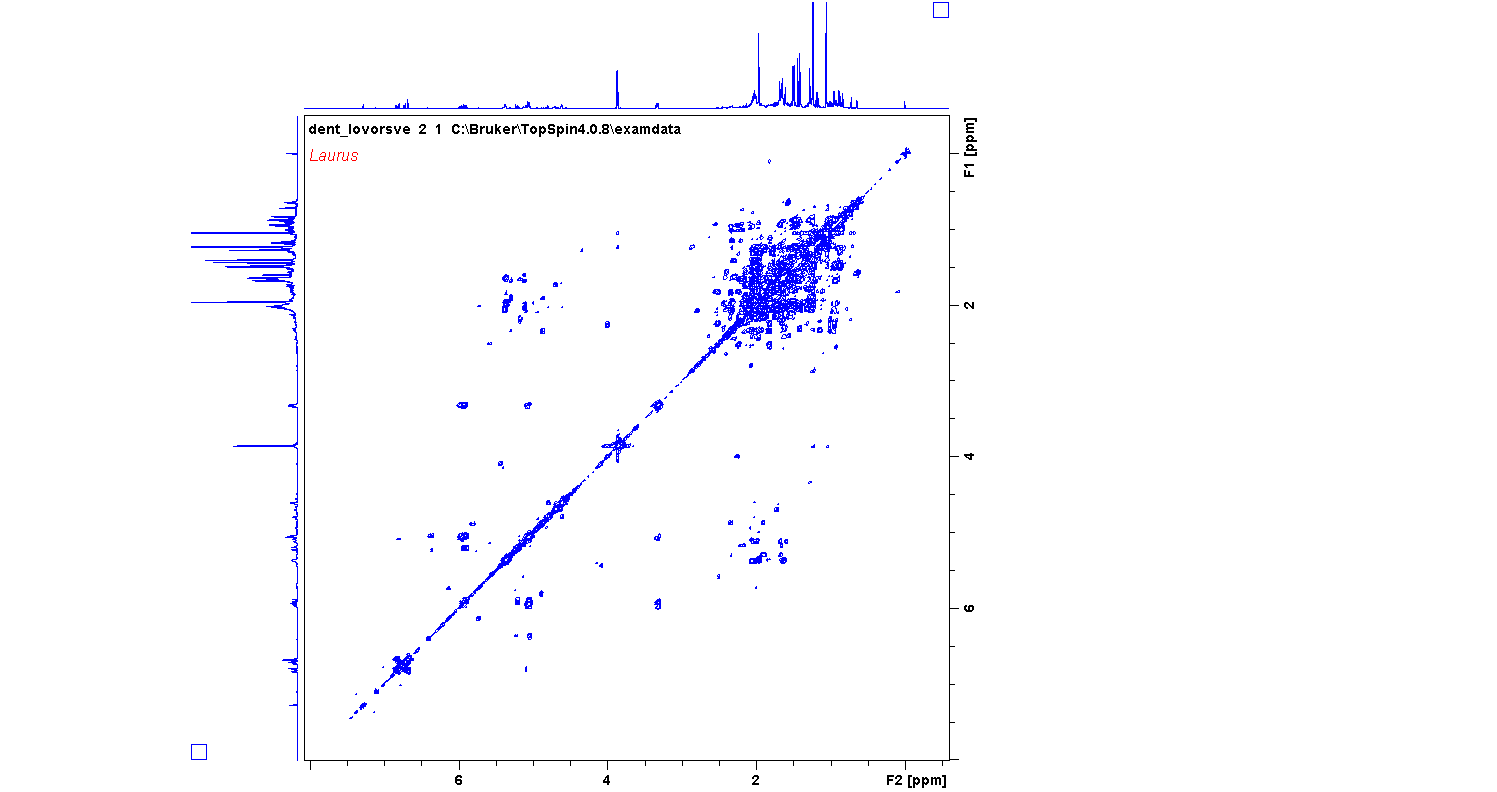


**Figure 8.** Bay laurel ^1^H-^1^H COSY NMR spectrum (600 MHz, CDCl_3_-*d*, 25 °C).


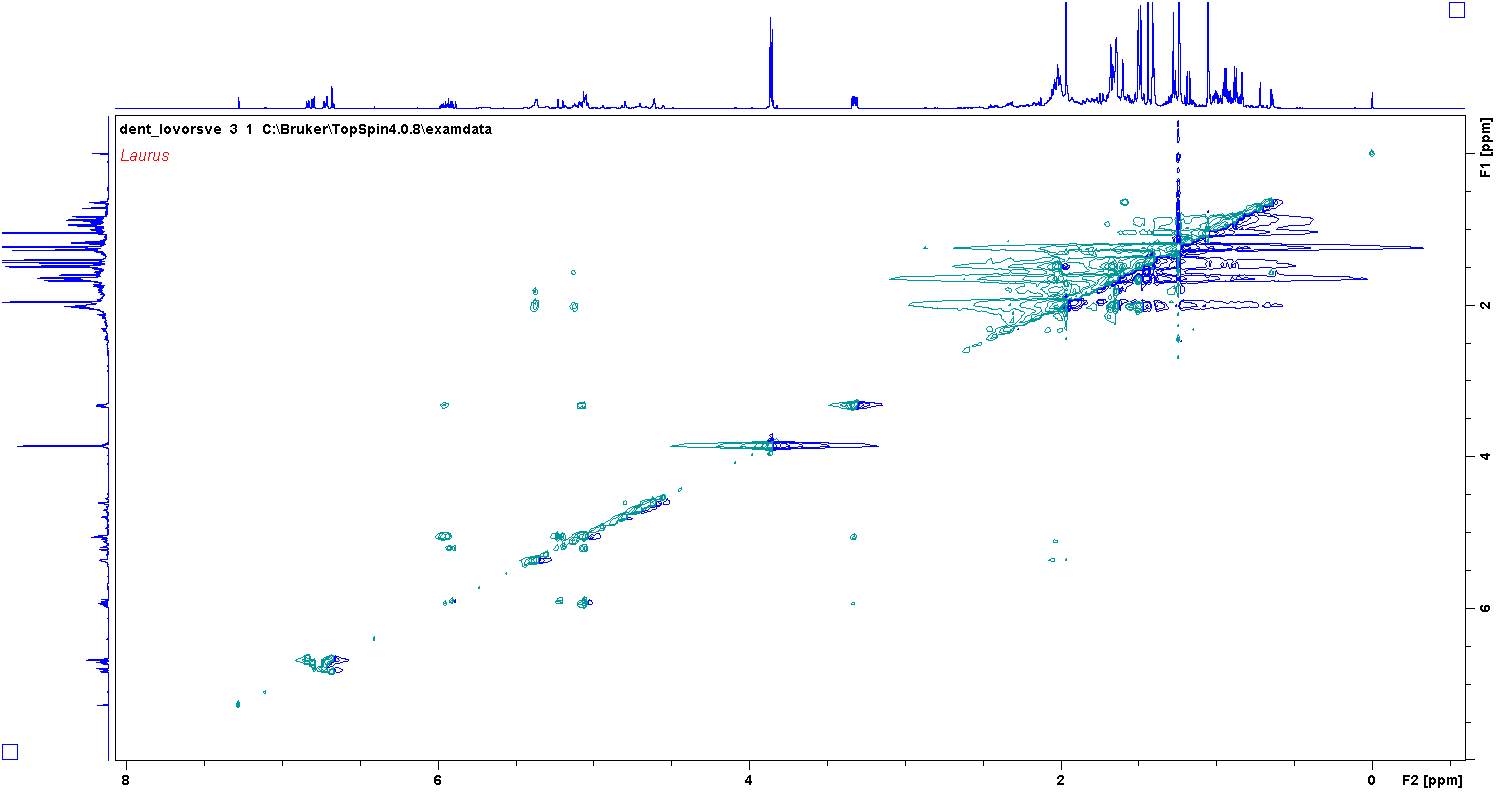


**Figure 9.** Bay laurel ^1^H-^1^H TOCSY NMR spectrum (600 MHz, CDCl_3_-*d*, 25 °C). .


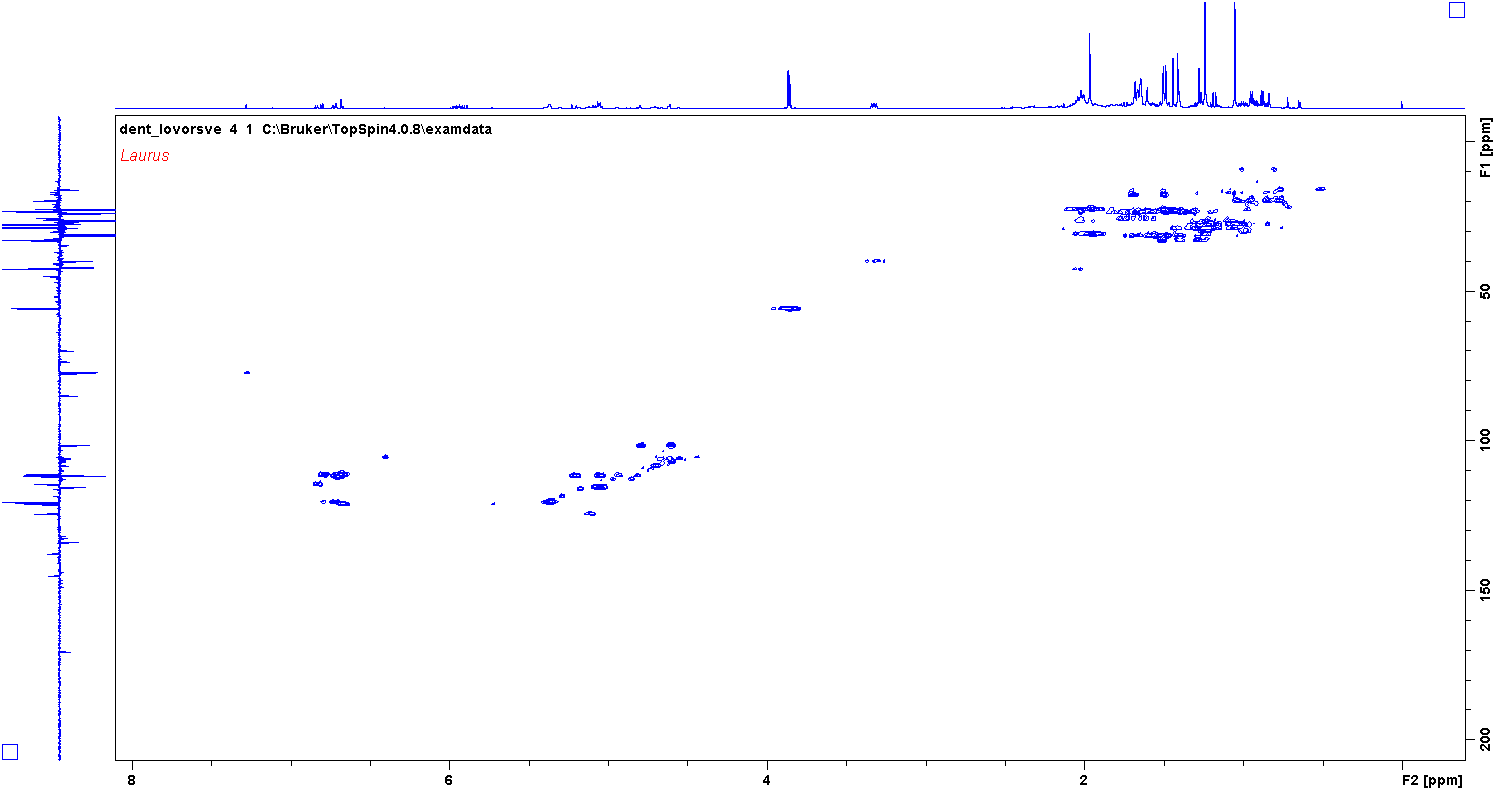


**Figure 10.** Bay laurel ^1^H-^13^C HMQC NMR spectrum (CDCl_3_-*d*, 25 °C). The 600 MHz ^1^H NMR spectrum is shown at the top edge and a 150 MHz ^13^C NMR spectrum at the left-hand edge.


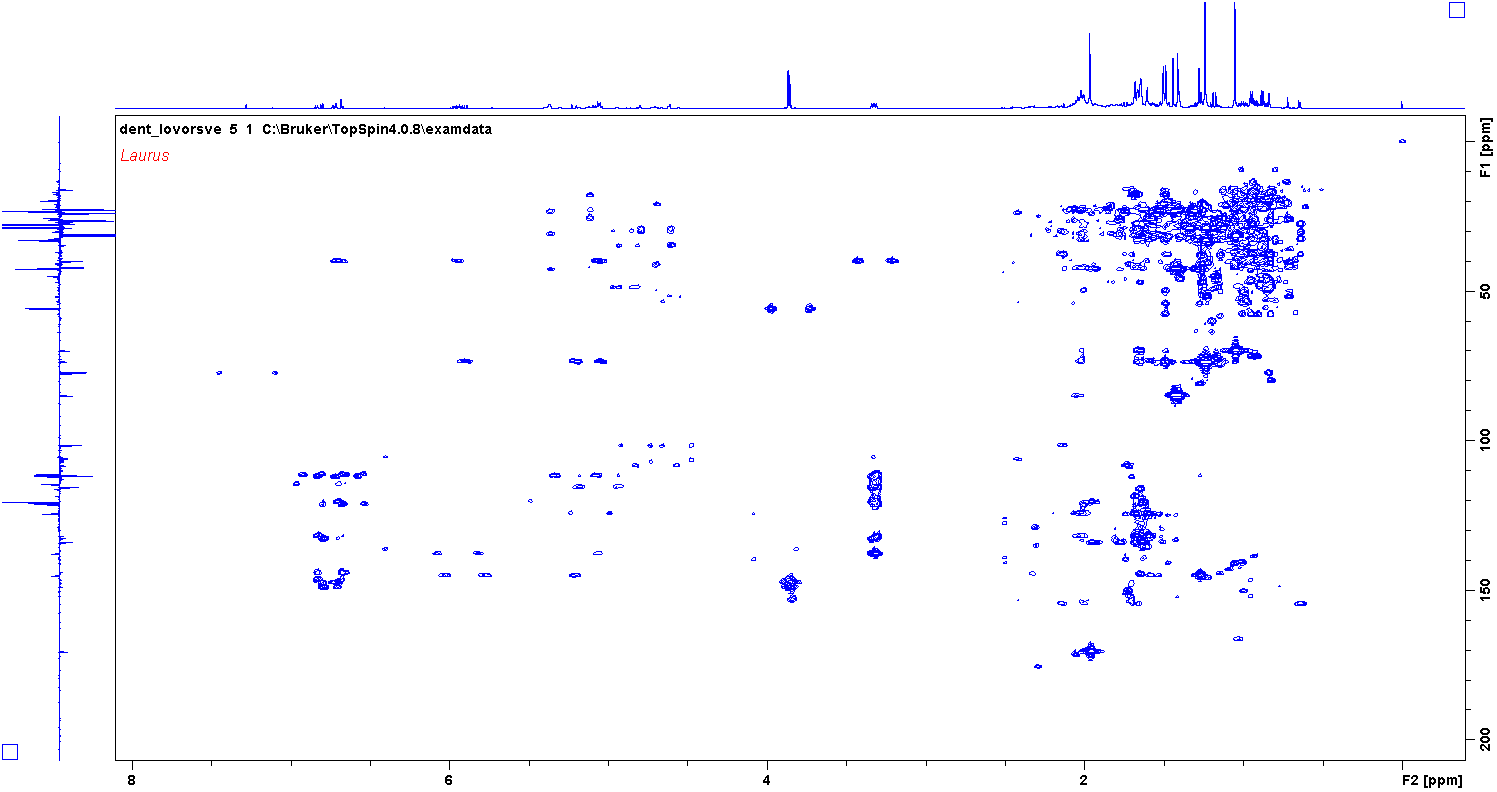


**Figure 11.** Bay laurel ^1^H-^13^C HMBC NMR spectrum (CDCl_3_-*d*, 25 °C). The 600 MHz ^1^H NMR spectrum is shown at the top edge and a 150 MHz ^13^C NMR spectrum at the left-hand edge.


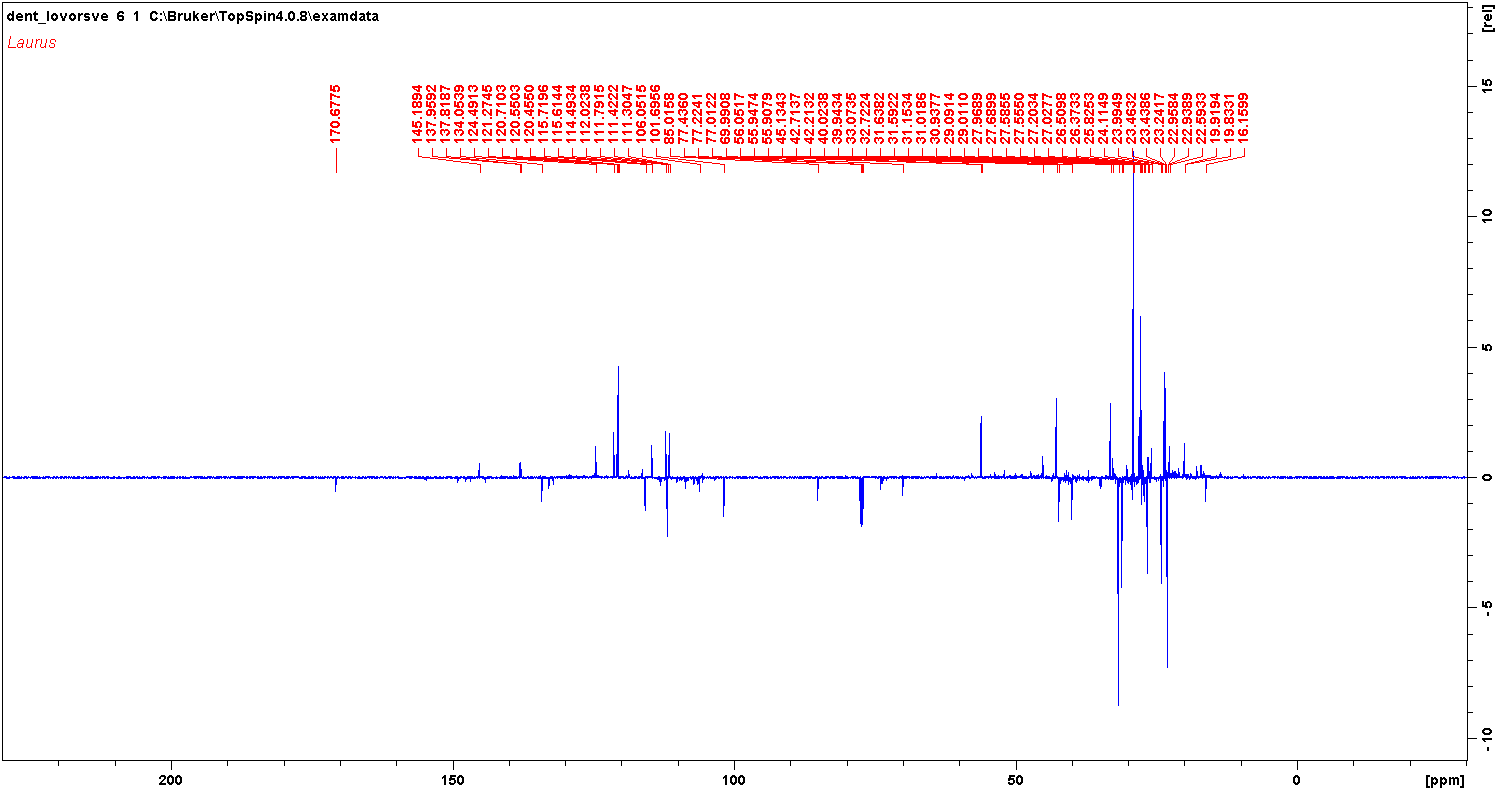


**Figure 12.** Bay laurel ^13^C APT NMR spectrum (150 MHz, 0.5 mL CDCl_3_; 5 mm sample tube; 25 °C; 64 *K* data points; *ca.* 30000 scans; 0.60 Hz/point; 1 s delay).


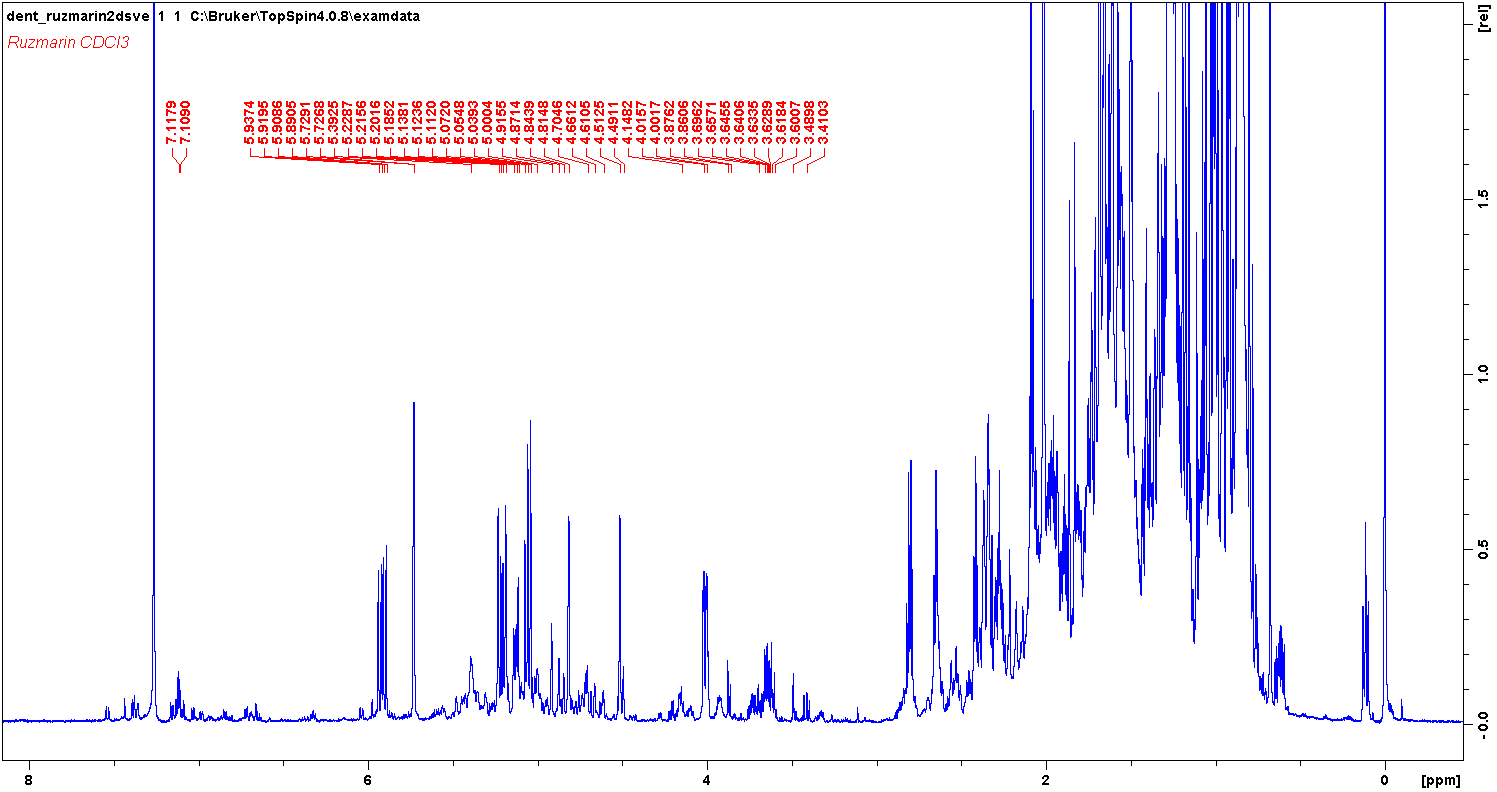

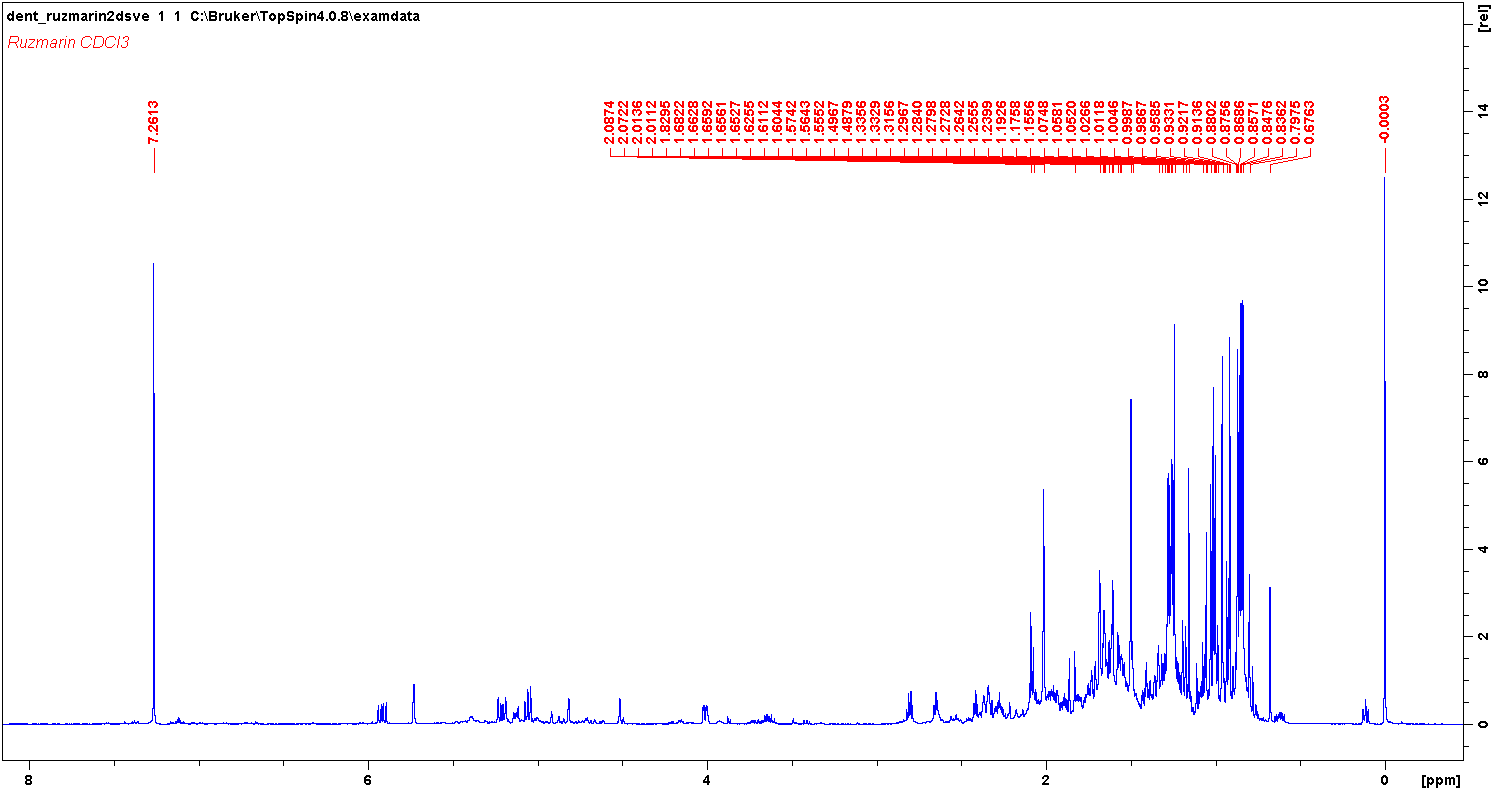


X6

**Figure 13.** Rosemary ^1^H NMR spectrum (600 MHz, 0.5 mL CDCl_3_; 5 mm sample tube; 25 °C; 32 *K* data points; 128 scans; 0.37 Hz/point; 1 s delay). .


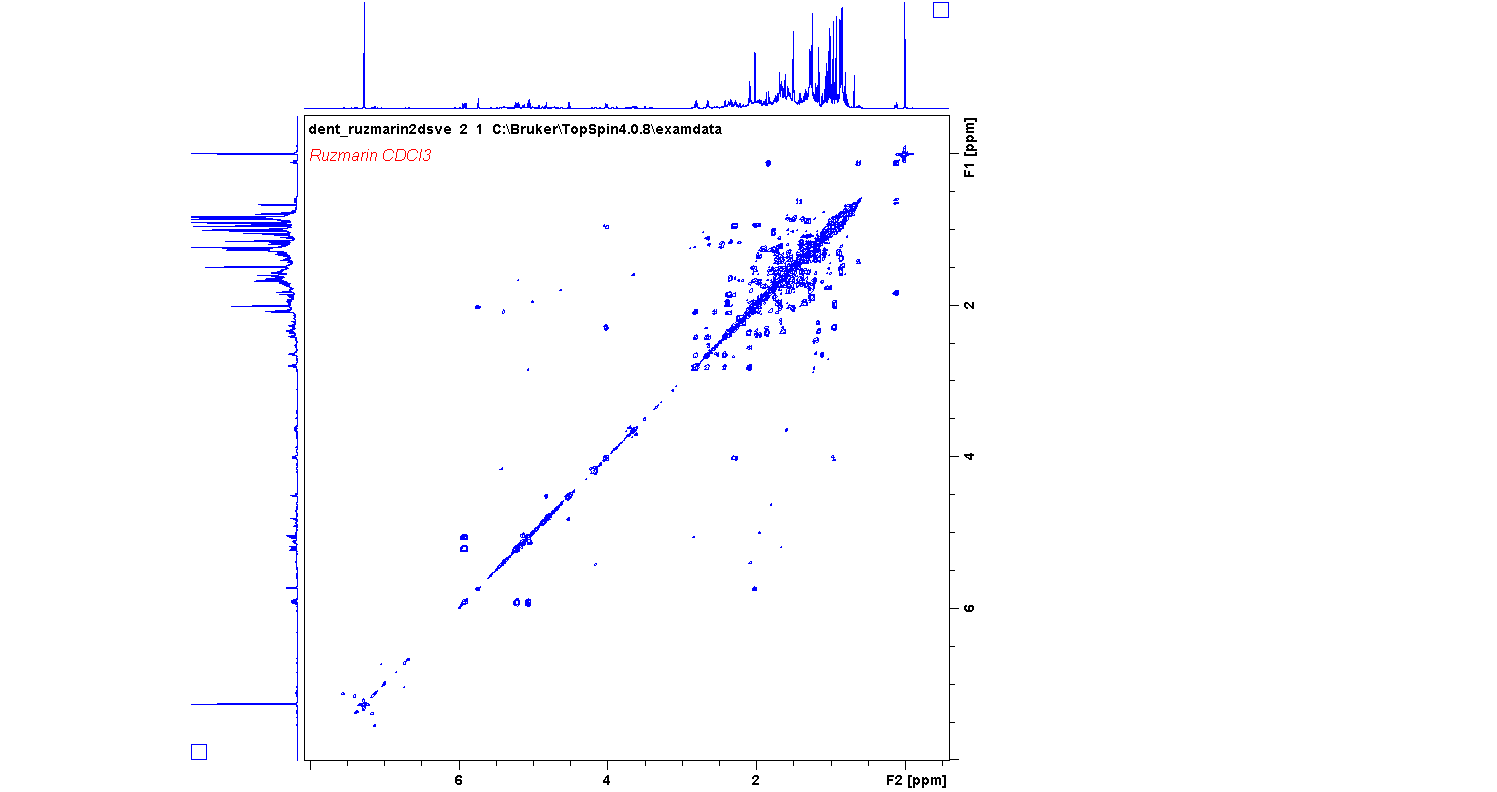


**Figure 14.** Rosemary ^1^H-^1^H COSY spectrum (600 MHz, CDCl_3_-*d*, 25 °C).


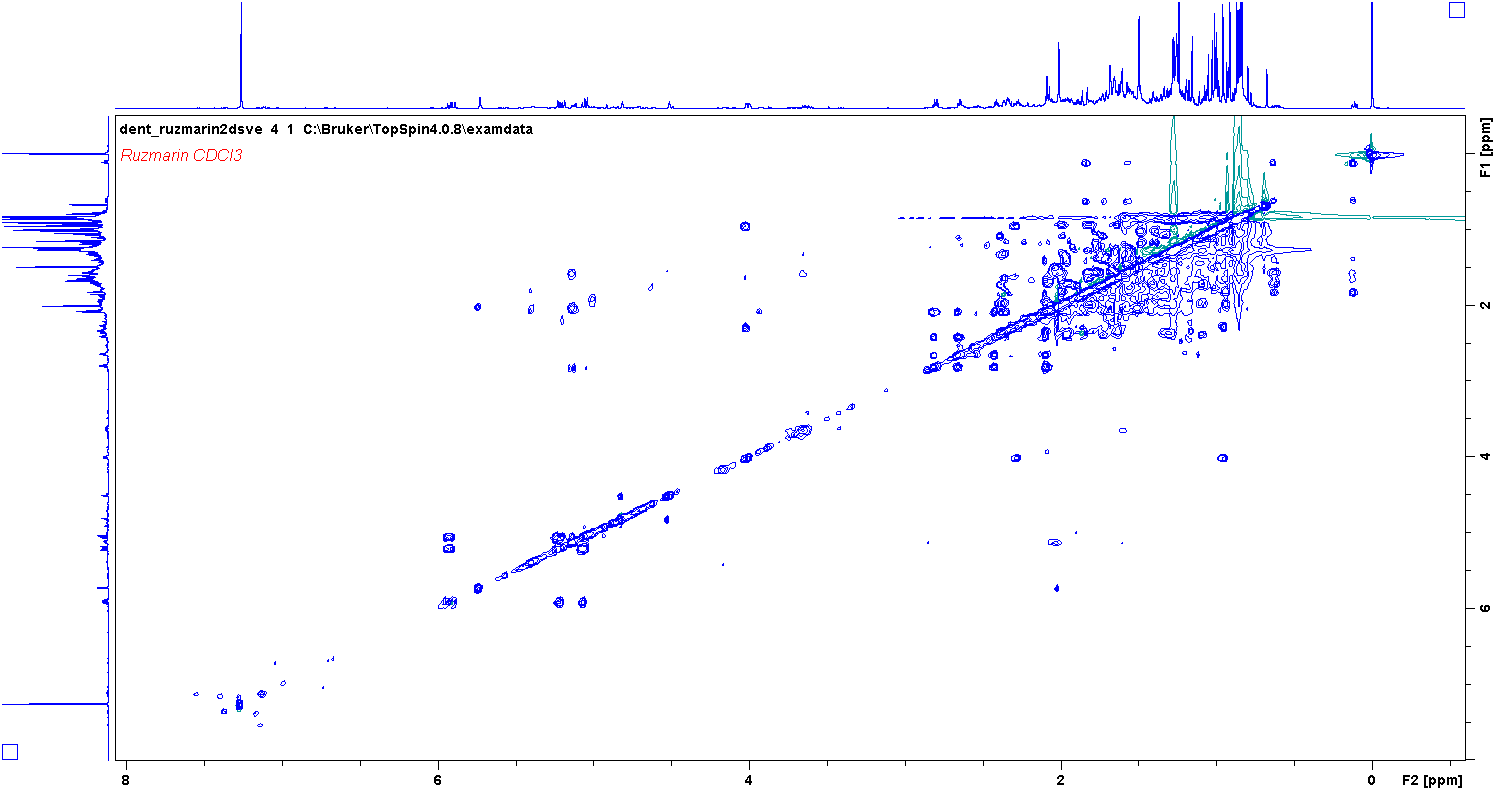


**Figure 15.** Rosemary ^1^H-^1^H TOCSY spectrum (600 MHz, CDCl_3_-*d*, 25 °C). .


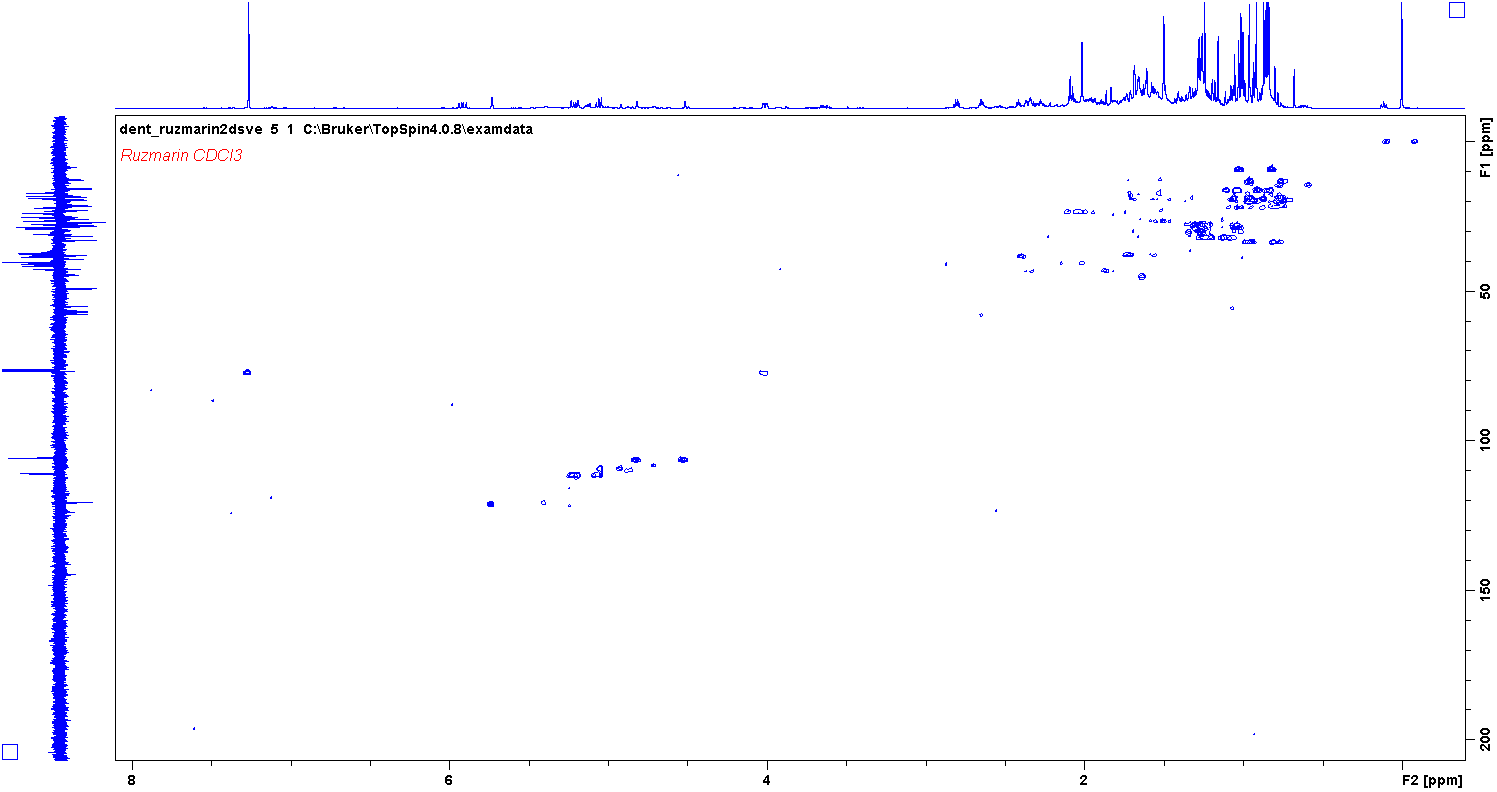


**Figure 16.** Rosemary ^1^H-^13^C HMQC spectrum (CDCl_3_-*d*, 25 °C). The 600 MHz ^1^H NMR spectrum is shown at the top edge and a 150 MHz ^13^C NMR spectrum at the left-hand edge.


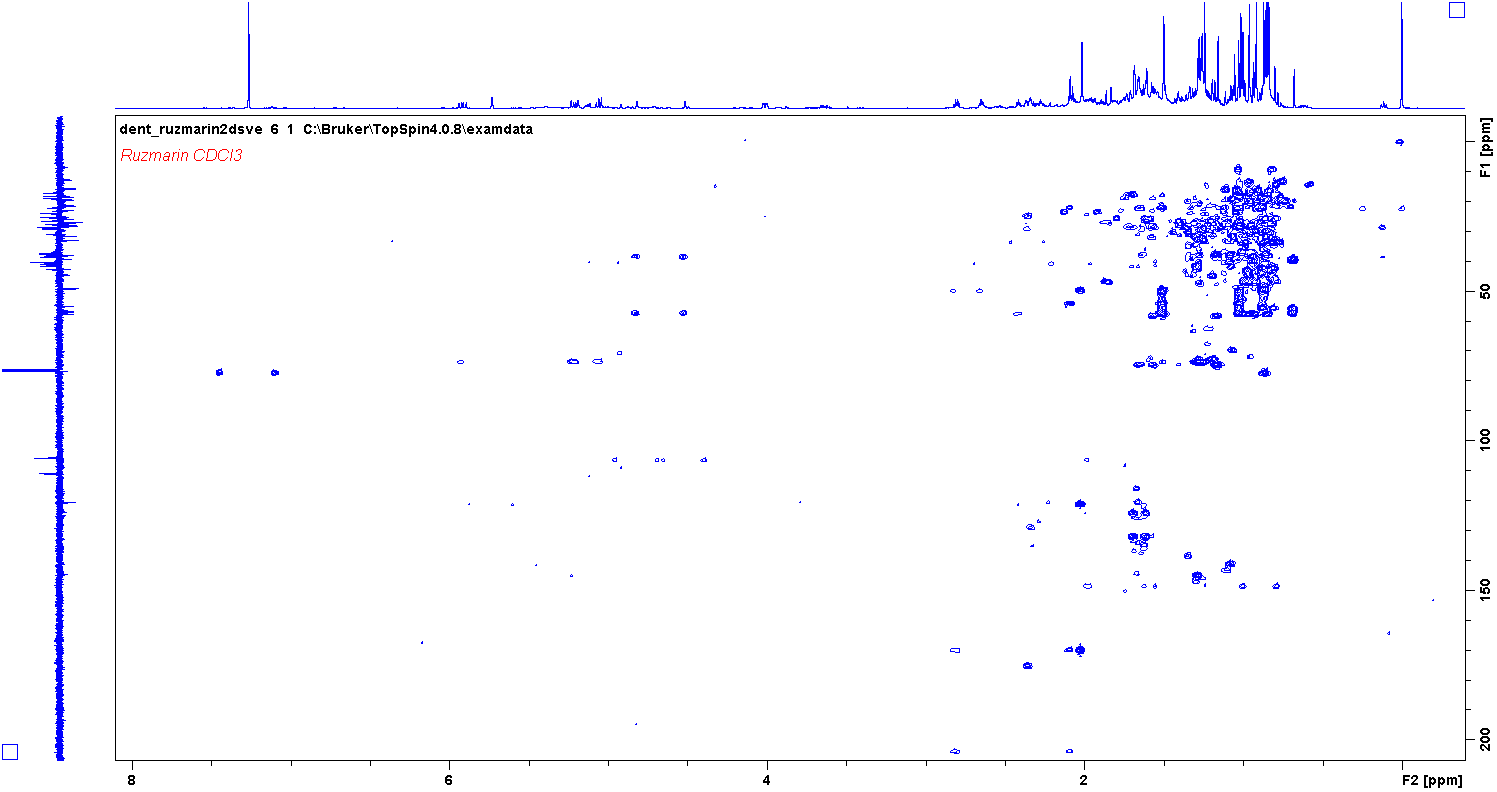


**Figure 17.** Rosemary ^1^H-^13^C HMBC spectrum (CDCl_3_-*d*, 25 °C). The 600 MHz ^1^H NMR spectrum is shown at the top edge and a 150 MHz ^13^C NMR spectrum at the left-hand edge.


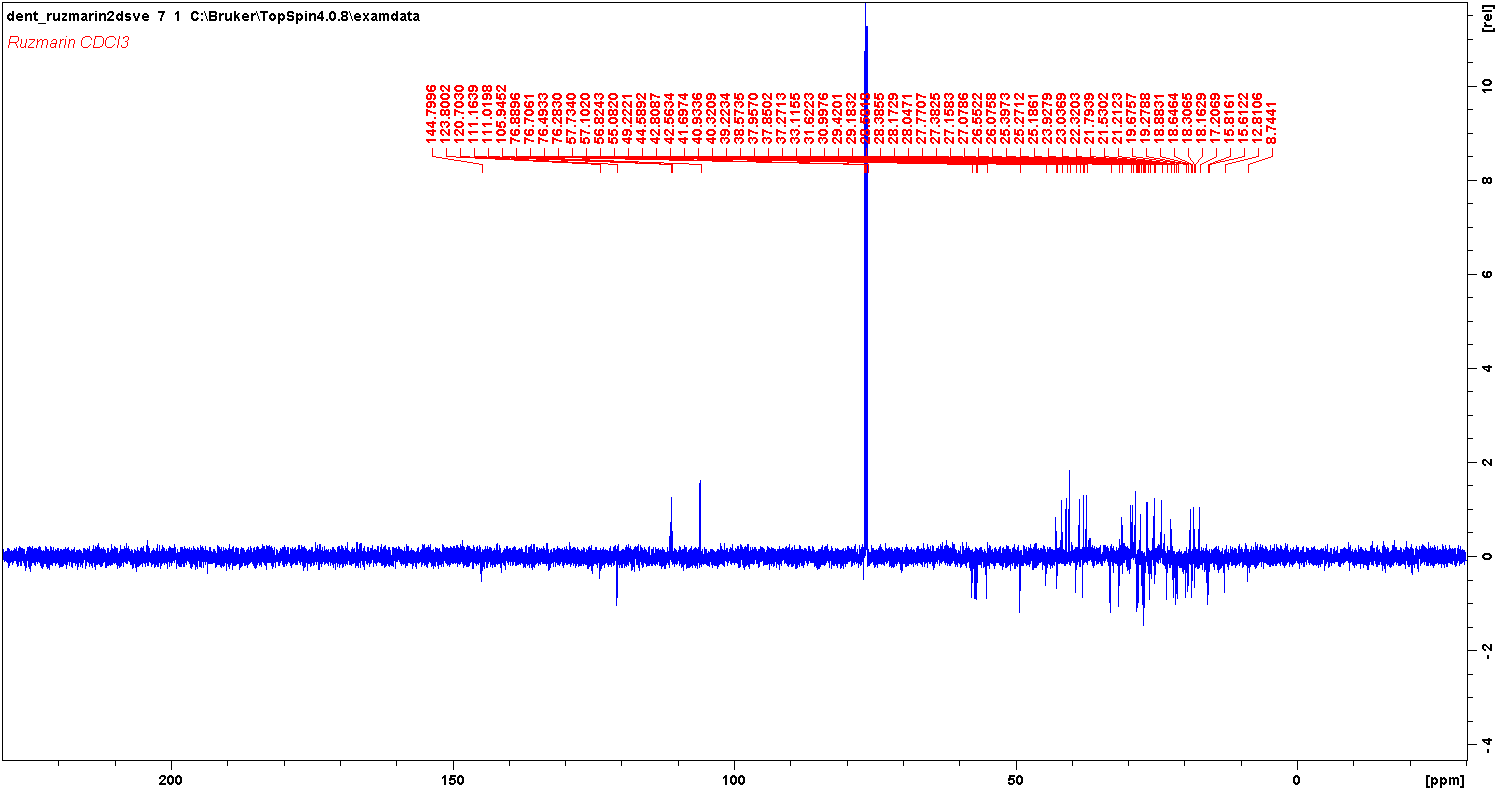


**Figure 18.** Rosemary ^13^C APT NMR spectrum (150 MHz, 0.5 mL CDCl_3_; 5 mm sample tube; 25 °C; 64 *K* data points; *ca.* 34000 scans; 0.60 Hz/point; 1 s delay).


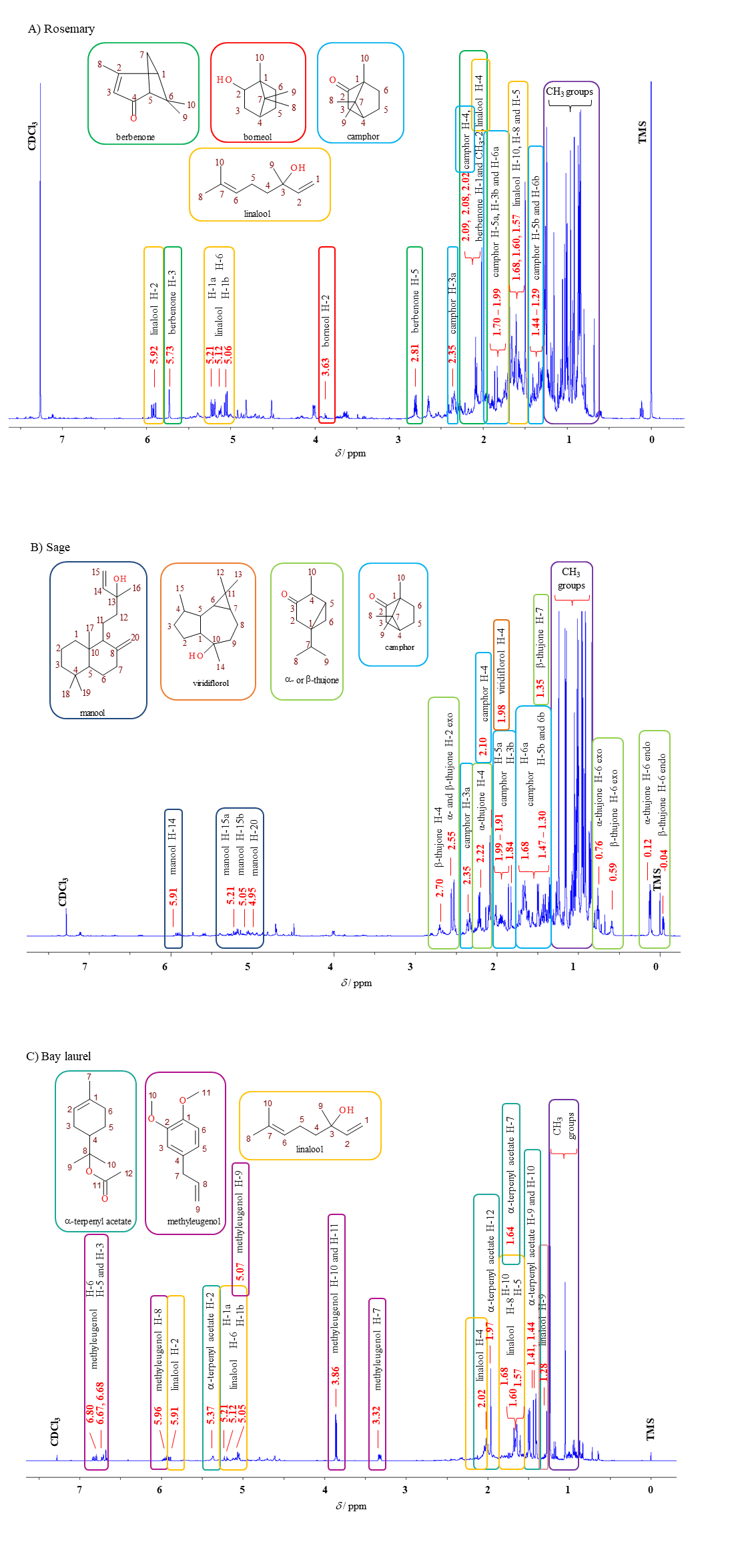


**Figure 19.** A) Rosemary, B) sage and C) bay laurel essential oils ^1^H NMR spectra at 600 MHz in CDCl_3_-*d*. Enumeration scheme used for the assignment of the NMR spectra is shown for every compound.
