# Supplementary material for: Effect of Enzymatic, Ultrasound, and Reflux Extraction Pretreatments on the Chemical Composition of Essential Oils
Source: Molecules. 2020 Oct 20;25(20):4818. doi: 10.3390/molecules25204818 (PMC7587977; doi:10.3390/molecules25204818)
Supplement: Supplementary file 1 [file molecules-25-04818-s001.zip › Supplementary file 2-molecules-942617-R2.docx]

Supplementary file 2 of the manuscript:

Effect of Enzymatic, Ultrasound, and Reflux Extraction Pretreatments on the Chemical Composition of Essential Oils

**Anđela Miljanović ^1^, Ana Bielen ^1,^*, Dorotea Grbin ^1^, Zvonimir Marijanović ^2^, Martina Andlar ^1^, Tonči Rezić ^1^, Sunčica Roca ^3^, Igor Jerković ^2^, Dražen Vikić-Topić ^3,4^ and Maja Dent ^1,^***

^1^ Faculty of Food Technology and Biotechnology, University of Zagreb, Pierottijeva 6, 10 000 Zagreb, Croatia, [amiljanovic@pbf.hr (A.M.)](mailto:amiljanovic@pbf.hr(A.M.)); [dorotea.polo@gmail.com](mailto:dorotea.polo@gmail.com) (D.G.); [martina.andlar@gmail.com](mailto:martina.andlar@gmail.com) (M.A.); [trezic@pbf.hr](mailto:trezic@pbf.hr); [maja.dent@pbf.unizg.hr](mailto:mfeges@pbf.hr) (T.R.)

^2^ Faculty of Chemistry and Technology, University of Split, Ruđera Boškovića 35, 21 000 Split, Croatia, [zmarijanovic@ktf-split.hr](mailto:zmarijanovic@ktf-split.hr) (Z.M.) ; [igor@ktf-split.hr](mailto:igor@ktf-split.hr) (I.J.)

^3^ NMR Centre, Ruđer Bošković Institute, Bijenička cesta 54, 10 000 Zagreb, Croatia, [sroca@irb.hr](mailto:sroca@irb.hr) (S.R.); [vikic@irb.hr](mailto:dvikic@irb.hr) (D.V.-T.)

^4^ Department of Natural and Health Sciences, Juraj Dobrila University of Pula, Zagrebačka 30, 52 100 Pula, Croatia

***** Correspondence: [abielen@pbf.hr](mailto:abielen@pbf.hr); Tel: +385 98 179 3307 (A.B.); [maja.dent@pbf.unizg.hr](mailto:mfeges@pbf.hr); Tel: +385 91 444 0555 (M.D.)

Academic Editor: Petras Rimantas Venskutonis

Received: 8 September 2020; Accepted: 19 October 2020; Published: date


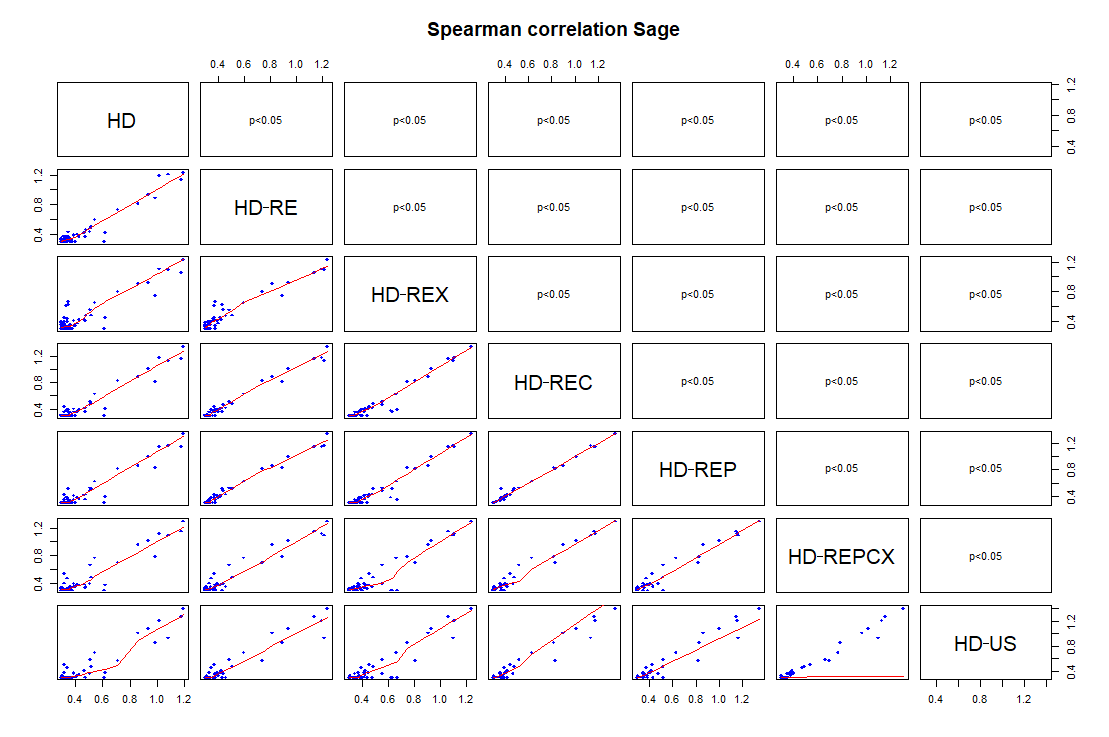


**Figure 20.** Scatter plot showing correlations between different pretreatments (on the diagonal) regarding chemical composition of sage essential oils. Significant p-values based on Spearman’s rank test are shown above the diagonal, while bivariate scatter plots are shown below the diagonal.


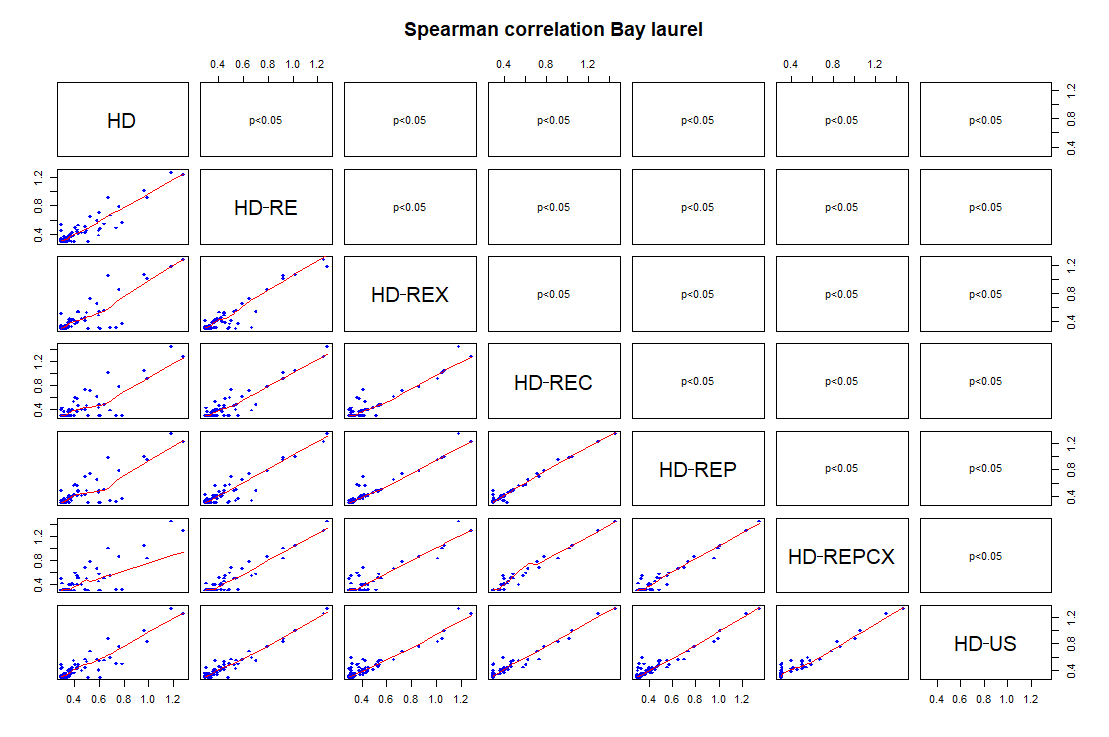


**Figure 21.** Scatter plot showing correlations between different pretreatments (on the diagonal) regarding chemical composition of bay laurel essential oils. Significant p-values based on Spearman’s rank test are shown above the diagonal, while bivariate scatter plots are shown below the diagonal.


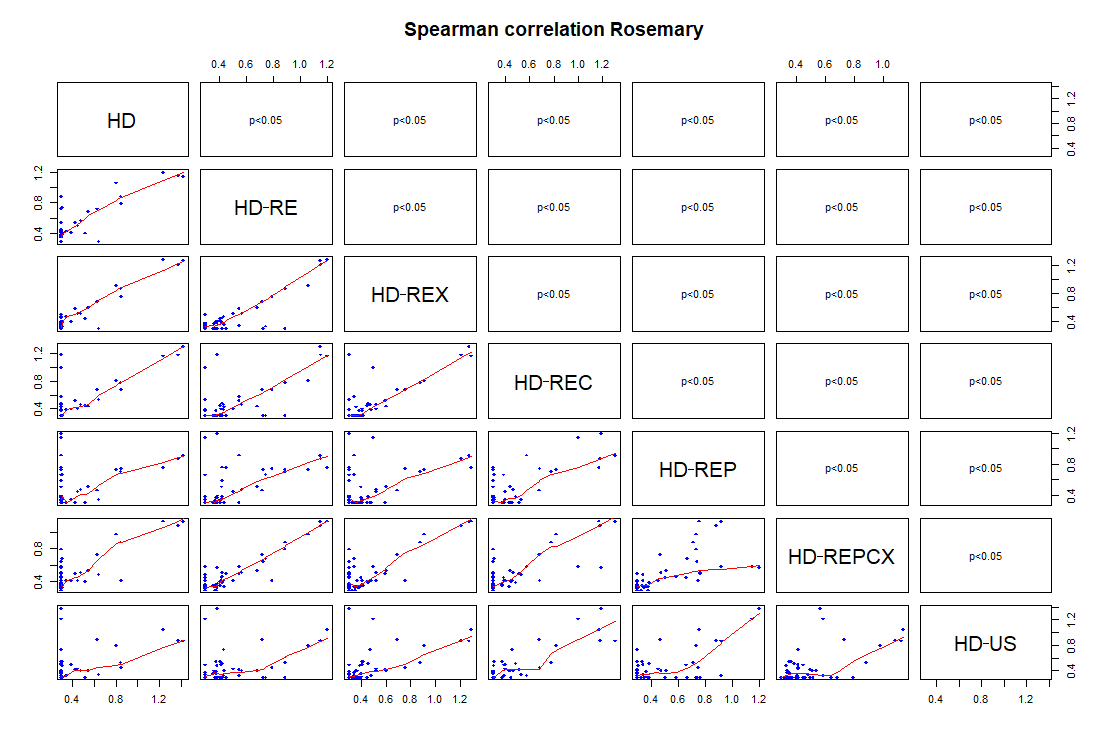


**Figure 22.** Scatter plot showing correlations between different pretreatments (on the diagonal) regarding chemical composition of rosemary essential oils. Significant p-values based on Spearman’s rank test are shown above the diagonal, while bivariate scatter plots are shown below the diagonal.
