# Supplementary material for: Effect of Enzymatic, Ultrasound, and Reflux Extraction Pretreatments on the Chemical Composition of Essential Oils
Source: Molecules. 2020 Oct 20;25(20):4818. doi: 10.3390/molecules25204818 (PMC7587977; doi:10.3390/molecules25204818)
Supplement: Supplementary file 1 [file molecules-25-04818-s001.zip › Supplementary file 3-molecules-942617-R2.docx]

Supplementary file 3 of the manuscript:

Effect of Enzymatic, Ultrasound, and Reflux Extraction Pretreatments on the Chemical Composition of Essential Oils

**Anđela Miljanović ^1^, Ana Bielen ^1,^*, Dorotea Grbin ^1^, Zvonimir Marijanović ^2^, Martina Andlar ^1^, Tonči Rezić ^1^, Sunčica Roca ^3^, Igor Jerković ^2^, Dražen Vikić-Topić ^3,4^ and Maja Dent ^1,^***

^1^ Faculty of Food Technology and Biotechnology, University of Zagreb, Pierottijeva 6, 10 000 Zagreb, Croatia, [amiljanovic@pbf.hr (A.M.)](mailto:amiljanovic@pbf.hr(A.M.)); [dorotea.polo@gmail.com](mailto:dorotea.polo@gmail.com) (D.G.); [martina.andlar@gmail.com](mailto:martina.andlar@gmail.com) (M.A.); [trezic@pbf.hr](mailto:trezic@pbf.hr); [maja.dent@pbf.unizg.hr](mailto:mfeges@pbf.hr) (T.R.)

^2^ Faculty of Chemistry and Technology, University of Split, Ruđera Boškovića 35, 21 000 Split, Croatia, [zmarijanovic@ktf-split.hr](mailto:zmarijanovic@ktf-split.hr) (Z.M.) ; [igor@ktf-split.hr](mailto:igor@ktf-split.hr) (I.J.)

^3^ NMR Centre, Ruđer Bošković Institute, Bijenička cesta 54, 10 000 Zagreb, Croatia, [sroca@irb.hr](mailto:sroca@irb.hr) (S.R.); [vikic@irb.hr](mailto:dvikic@irb.hr) (D.V.-T.)

^4^ Department of Natural and Health Sciences, Juraj Dobrila University of Pula, Zagrebačka 30, 52 100 Pula, Croatia

***** Correspondence: [abielen@pbf.hr](mailto:abielen@pbf.hr); Tel: +385 98 179 3307 (A.B.); [maja.dent@pbf.unizg.hr](mailto:mfeges@pbf.hr); Tel: +385 91 444 0555 (M.D.)

Academic Editor: Petras Rimantas Venskutonis

Received: 8 September 2020; Accepted: 19 October 2020; Published: date

**Table 1.** Chemical composition of sage essential oils isolated by hydrodistillation with and without different pretreatments.

|  | **group** | **compound** | **RI*^1^*** | **% total peak area** | | | | | | |
| --- | --- | --- | --- | --- | --- | --- | --- | --- | --- | --- |
| **monoterpenes** |  |  |  | HD^2^ | HD-RE^3^ | HD-REX^4^ | HD-REC^5^ | HD-REP^6^ | HD-REPCX^7^ | HD-US^8^ |
|  | monoterpene hydrocarbons | *cis*-salvene | < 900 | 0.01 | 0.01 | 0.08 | 0.02 | 0.03 | 0.15 | 0.01 |
|  |  | tricyclene | 929 | 0.03 | 0.04 | 0.01 | 0.01 | 0.01 | 0.10 | 0.01 |
|  |  | α-thujene | 932 | 0.01 | 0.01 | 0.01 | 0.01 | 0.01 | 0.01 | 0.01 |
|  |  | α-pinene^S^ | 941 | 1.25 | 1.03 | 1.56 | 1.30 | 1.35 | 2.64 | 1.83 |
|  |  | camphene^S^ | 956 | 1.49 | 1.94 | 2.54 | 2.26 | 2.26 | 3.85 | 2.91 |
|  |  | verbenene | 962 | 0.04 | -^9^ | - | - | - | - | - |
|  |  | sabinene^S^ | 979 | 0.01 | 0.01 | 0.01 | 0.01 | 0.01 | 0.01 | 0.01 |
|  |  | β-pinene^S^ | 982 | 0.45 | 0.47 | 0.59 | 0.53 | 0.59 | 0.46 | 0.40 |
|  |  | β-myrcene^S^ | 992 | 0.08 | 0.12 | 0.34 | 0.28 | 0.25 | 0.53 | 0.37 |
|  |  | δ-car-3-ene^S^ | 1014 | 0.05 | - | - | - | - | - | - |
|  |  | α-terpinene^S^ | 1021 | 0.01 | 0.01 | 0.02 | 0.01 | 0.01 | 0.09 | 0.01 |
|  |  | *p*-cymene^S^ | 1030 | 0.19 | 0.22 | 0.47 | 0.46 | 0.39 | 0.99 | 0.90 |
|  |  | limonene^S^ | 1034 | 0.10 | 0.32 | 0.85 | 0.77 | 0.68 | 1.46 | 1.16 |
|  |  | γ-terpinene^S^ | 1063 | 0.03 | 0.01 | 0.12 | 0.02 | 0.01 | 0.08 | 0.01 |
|  |  | α-terpinolene | 1091 | 0.06 | 0.01 | 0.01 | 0.01 | 0.02 | 0.05 | 0.01 |
|  | oxygenated monoterpenes | 1,8-cineole^S^ | 1038 | 5.22 | 4.56 | 6.09 | 5.94 | 5.31 | 7.23 | 8.22 |
|  |  | linalool^S^ | 1104 | 2.17 | 0.61 | 0.79 | 0.59 | 0.51 | 0.37 | 0.39 |
|  |  | α-thujone*^S^ | 1110 | 13.52 | 15.33 | 15.16 | 20.15 | 19.91 | 18.18 | 23.48 |
|  |  | β-thujone*^S^ | 1121 | 6.57 | 6.63 | 6.45 | 8.33 | 8.03 | 8.51 | 10.22 |
|  |  | chrysanthenone | 1129 | 0.21 | - | - | - | - | 0.04 | - |
|  |  | thujyl alcohol | 1141 | 0.45 | 0.38 | 0.19 | 0.28 | 0.25 | 0.23 | 0.27 |
|  |  | camphor*^S^ | 1149 | 12.99 | 11.69 | 9.50 | 12.58 | 11.98 | 12.43 | 17.03 |
|  |  | pinocarvone | 1167 | 0.07 | - | - | - | - | - | - |
|  |  | borneol^S^ | 1172 | 7.73 | 5.77 | 3.52 | 4.59 | 4.76 | 4.12 | 5.18 |
|  |  | *trans*-pinocarvone | 1179 | 0.33 | - | - | - | - | - | - |
|  |  | 4-terpineol^S^ | 1182 | 0.92 | 0.65 | 0.65 | 0.61 | 0.63 | 0.44 | 0.53 |
|  |  | *p*-cymen-8-ol^S^ | 1191 | 0.28 | 0.18 | 0.01 | 0.01 | 0.01 | 0.12 | 0.01 |
|  |  | α-terpineol^S^ | 1195 | 0.99 | 0.31 | 0.58 | 0.32 | 0.28 | 0.18 | 0.01 |
|  |  | myrtenol^S^ | 1200 | 0.37 | 0.13 | - | - | - | 0.05 | - |
|  |  | homomyrtenol | 1208 | 0.39 | - | - | - | - | - | - |
|  |  | *trans*-carveol | 1224 | 0.17 | 0.11 | 0.01 | 0.01 | 0.01 | 0.07 | - |
|  |  | geraniol^S^ | 1261 | 0.11 | 0.01 | - | - | - | - | - |
|  |  | bornyl acetate^S^ | 1287 | 1.31 | 1.20 | 1.04 | 1.04 | 1.30 | 1.09 | 1.00 |
|  |  | *trans*-sabinyl acetate | 1294 | 0.61 | 0.51 | 0.37 | 0.34 | 0.44 | 0.39 | 0.16 |
|  |  | *trans*-carvyl acetate | 1341 | 0.04 | - | - | - | - | - | - |
|  |  | α-terpenyl acetate | 1355 | 0.25 | 0.68 | 2.25 | 0.34 | 1.32 | - | - |
|  | **total monoterpenes** |  |  | **58.51** | **52.95** | **53.22** | **60.82** | **60.36** | **63.78** | **74.23** |
| sesquiterpenes | sesquiterpene hydrocarbons | α-ylangene | 1373 | 0.06 | - | - | 0.01 |  | 0.04 | - |
|  |  | α-copaene^S^ | 1378 | 0.18 | - | 0.12 | 0.02 | 0.01 | 0.11 | - |
|  |  | β-elemene^S^ | 1393 | - | - | 0.26 | 0.01 | 0.01 | - | - |
|  |  | *trans*-caryophyllene^S^ | 1421 | 1.21 | 0.73 | 1.59 | 0.93 | 1.03 | 0.51 | 0.30 |
|  |  | α-cadinene | 1540 | 0.04 | 0.01 | 0.01 | 0.01 | 0.01 | 0.07 | 0.01 |
|  |  | α-guaiene | 1441 | 0.01 | 0.01 | 0.22 | 0.03 | 0.03 | 0.01 | 0.01 |
|  |  | α-humulene^S^ | 1456 | 3.15 | 3.44 | 4.40 | 4.78 | 4.60 | 3.00 | 1.76 |
|  |  | alloaromadendrene^S^ | 1462 | 0.06 | 0.01 | 0.01 | 0.03 | 0.01 | 0.08 | 0.01 |
|  |  | α-amorphene | 1478 | 0.32 | 0.19 | 0.28 | 0.27 | 0.20 | 0.25 | 0.01 |
|  |  | β-selinene | 1488 | 0.05 | - | 0.16 | 0.01 | - | - | - |
|  |  | ledene^S^ | 1496 | - | - | 0.43 | 0.05 | - | 0.16 | - |
|  |  | α-muurolene | 1501 | 0.13 | - | 0.18 | 0.01 | - | 0.07 | - |
|  |  | β-bisabolene | 1510 | 0.07 | - | - | - | - | - | - |
|  |  | γ-cadinene | 1515 | 0.26 | 0.07 | 0.19 | 0.10 | 0.09 | 0.10 | 0.01 |
|  |  | δ-cadinene^S^ | 1525 | 0.67 | 0.35 | 0.62 | 0.45 | 0.35 | 0.37 | 0.21 |
|  |  | *trans*-α-bisabolene | 1545 | - | 0.01 | - | - | - | - | - |
|  |  | α-calacorene | 1546 | 0.08 | 0.01 | 0.17 | 0.01 | 0.01 | 0.01 | - |
|  | oxyganeted sesquiterpenes | berbenone | 1212 | 2.10 | 0.01 | - | - | - | - | - |
|  |  | spathulenol | 1581 | - | 0.10 | 0.50 | - | 0.01 | - | - |
|  |  | caryophyllene oxide^S^ | 1584 | 1.00 | 0.86 | 1.01 | 0.62 | 0.71 | 0.25 | - |
|  |  | veridiflorol* | 1594 | 10.13 | 14.39 | 10.60 | 11.70 | 12.63 | 10.64 | 6.52 |
|  |  | α-caryophylladienol | 1640 | 0.31 | - | 0.29 | 0.05 | 0.01 | - | - |
|  |  | α-cadinol | 1646 | - | 0.16 | - | 0.01 | - | 0.09 | - |
|  |  | β-eudesmol^S^ | 1654 | 0.10 | 0.18 | 0.46 | 0.02 | - | 0.06 | - |
|  |  | t-muurolol | 1658 | 0.19 | 0.27 | 0.74 | 0.01 |  | 0.09 | - |
|  |  | (*E*,*E*)-farnesyl acetone | 1919 | 0.15 | - | - | - | - | - | - |
|  |  | manool* | 2055 | 8.30 | 13.74 | 10.80 | 12.99 | 12.24 | 11.47 | 14.33 |
|  | **total sesquiterpenes** |  |  | **28.57** | **34.54** | **33.04** | **32.12** | **31.95** | **27.38** | **23.17** |
| others | phenylpropane derivatives | thymol^S^ | 1296 | 0.04 | 0.36 | 0.01 | 0.01 | 0.01 | 0.06 | 0.01 |
|  |  | carvacrol^S^ | 1307 | 0.50 | 0.01 | 0.17 | 0.02 | 0.01 | 0.26 | 0.01 |
|  |  | eugenol^S^ | 1363 | 0.23 | 0.36 | 2.68 | 0.47 | 0.23 | - | - |
|  |  | methyleugenol^S^ | 1409 | 0.15 | 0.34 | 2.14 | 0.37 | 0.45 | - | - |
|  |  | elemicin | 1561 | - | - | 0.27 | - | - | - | - |
|  | other compounds | methyl jasmonate^S^ | 1651 | 0.11 | - | - | - | - | - | - |
|  |  | pentadecanal^S^ | 1715 | 0.03 | - | - | - | - | - | - |
|  |  | hexadecan-1-ol^S^ | 1885 | 0.01 | - | 0.01 | - | - | - | - |
|  | **total others** |  |  | **1.07** | **1.07** | **5.28** | **0.87** | **0.7** | **0.32** | **0.02** |
|  | **total:** |  |  | **88.15** | **88.56** | **91.54** | **93.81** | **93.01** | **91.48** | **97.42** |

^1^Retention index on a MSD model 5975 C and HP-5MS; ^2^HD - hydrodistillation without pretreatment (negative control); ^3^HD-RE - hydrodistillation with reflux extraction pretreatment; hydrodistillation with reflux extraction pretreatment assisted with enzymes: ^4^HD-REX xylanase; ^5^HD-REC cellulase; ^6^HD-REP pectinase; ^7^HD-REPCX pectinase + cellulase + xylanase; ^8^HD-US hydrodistillation with ultrasonic pretreatment; ^9^(-) not detected; * - compounds confirmed by NMR*,* ^S^ - compounds identified with the standard.

**Table 2.** Chemical composition of bay laurel essential oils isolated by hydrodistillation with and without different pretreatments.

|  | **Group** | **Compound** | **RI^1^** | **% Total Peak Area** | | | | | | |
| --- | --- | --- | --- | --- | --- | --- | --- | --- | --- | --- |
| **monoterpes**  **nes** |  |  |  | HD^2^ | HD-RE^3^ | HD-REX^4^ | HD-REC^5^ | HD-REP^6^ | HD-REPCX^7^ | HD-US^8^ |
|  | monoterpene hydrocarbons | α-thujene | 932 | 0.04 | 0.04 | 0.02 | 0.01 | 0.14 | 0.01 | 0.09 |
|  |  | α-pinene^S^ | 941 | 0.56 | 1.12 | 0.32 | 2.05 | 1.77 | 1.88 | 1.10 |
|  |  | camphene^S^ | 956 | 0.28 | 0.21 | 0.08 | 0.23 | 0.25 | 0.02 | 0.23 |
|  |  | sabinene^S^ | 979 | 1.08 | 1.23 | 0.60 | 3.39 | 3.03 | 2.74 | 1.74 |
|  |  | β-pinene^S^ | 982 | 0.66 | 0.89 | 0.41 | 1.82 | 1.63 | 1.47 | 0.84 |
|  |  | β-myrcene^S^ | 992 | 0.11 | 0.04 | 0.03 | 0.01 | 0.19 | 0.02 | 0.12 |
|  |  | δ-car-3-ene^S^ | 1014 | -^9^ | 0.05 | - | - | 0.04 | 0.01 | 0.07 |
|  |  | α-terpinene^S^ | 1021 | 0.05 | 0.01 | 0.07 | 0.01 | 0.19 | 0.02 | 0.15 |
|  |  | p-cymene^S^ | 1030 | 0.14 | 0.09 | 0.08 | 0.01 | 0.21 | 0.04 | 0.32 |
|  |  | limonene^S^ | 1034 | 0.01 | 0.01 | 0.05 | 0.69 | 0.02 | 0.62 | 0.76 |
|  |  | γ-terpinene^S^ | 1063 | 0.08 | 0.07 | 0.13 | 0.32 | 0.32 | 0.02 | 0.28 |
|  |  | α-terpinolene | 1091 | 0.03 | - | 0.05 | - | 0.09 | - | 0.08 |
|  | oxygenated monoterpenes | 1,8-cineole | 1038 | 13.26 | 16.86 | 13.23 | 26.40 | 20.15 | 27.10 | 19.56 |
|  |  | *cis*-sabinene hydrate | 1073 | 0.10 | 0.33 | 0.04 | - | - | - | 0.22 |
|  |  | linalool*^S^ | 1104 | 7.80 | 6.39 | 8.30 | 6.27 | 7.14 | 4.91 | 4.92 |
|  |  | α-thujone^S^ | 1110 | 3.51 | 1.09 | 0.09 | 0.01 | 0.09 | 0.02 | 1.18 |
|  |  | β-thujone^S^ | 1121 | 1.97 | 0.45 | 0.06 | - | 0.01 | - | 0.53 |
|  |  | *cis*-p-menth-2-en-1-ol | 1127 | - | - | 0.09 | - | 0.05 | - | 0.06 |
|  |  | chrysanthenone | 1129 |  | 0.13 | - | - | - | - | 0.06 |
|  |  | thujyl alcohol | 1141 | 0.09 | - | - | - | - | - | - |
|  |  | terpenene-1-ol | 1146 | 0.11 | - | 0.15 | - | - | - | 0.07 |
|  |  | camphor^S^ | 1149 | 2.90 | 2.69 | 0.05 | 0.48 | 0.20 | 1.57 | 1.93 |
|  |  | pinocarvone | 1167 | - | 0.09 | - | - | - | - | 0.12 |
|  |  | borneol^S^ | 1172 | 2.04 | 3.05 | 1.45 | 1.09 | 1.06 | 1.74 | 1.55 |
|  |  | isopinocamphone | 1178 | - | 0.20 | - | - | - | - | - |
|  |  | 4-terpineol^S^ | 1182 | 1.83 | 1.93 | 2.55 | 2.23 | 2.46 | 2.65 | 1.71 |
|  |  | p-cymen-8-ol^S^ | 1191 | - | 0.08 | - | - | - | - | 0.05 |
|  |  | α-terpineol^S^ | 1195 | 3.76 | 4.25 | 5.26 | 4.06 | 4.17 | 5.27 | 3.84 |
|  |  | myrtenol^S^ | 1200 | - | 0.14 | 0.06 | - | - | - | - |
|  |  | homomyrtenol | 1208 | - | 0.21 | - | - | - | - | - |
|  |  | nerol^S^ | 1233 | 0.35 | 0.48 | 0.56 | 0.01 | 0.35 | 0.01 | 0.21 |
|  |  | linalyl acetate^S^ | 1259 | 0.29 | 0.07 | 0.12 | 0.01 | 0.03 | 0.01 | 0.02 |
|  |  | geraniol^S^ | 1261 | - | 0.10 | 0.09 | - | - | - | - |
|  |  | bornyl acetate^S^ | 1287 | 1.97 | 0.83 | 1.11 | 0.74 | 0.83 | 0.83 | 0.64 |
|  |  | *trans*-sabinyl acetate | 1294 | 0.15 | - | - | - | - | - |  |
|  |  | δ-terpinyl acetate | 1319 | 0.90 | 0.63 | 0.81 | 0.51 | 0.61 | 0.46 | 0.54 |
|  |  | 2-acetoxy-1,8-cineole | 1344 | 0.16 | 0.19 | 0.24 | 0.01 | 0.18 | 0.02 | 0.12 |
|  |  | α-terpenyl acetate* | 1355 | 16.94 | 15.71 | 17.18 | 17.69 | 15.16 | 18.16 | 15.84 |
|  |  | neryl acetate | 1367 | 0.23 | 0.13 | 0.23 | 0.01 | 0.18 | 0.01 | 0.12 |
|  |  | trans-cinnamyl acetate | 1449 | 0.20 | - | 0.15 | - | 0.12 | - | - |
|  |  | spathulenol | 1581 | 2.40 | 1.52 | 1.61 | 1.08 | 1.17 | 1.18 | 1.62 |
|  |  | manool | 2055 | 2.10 | 1.03 | - | - | - | - | - |
|  | **total monoterpenes** |  |  | **66.1** | **62.34** | **55.27** | **69.14** | **61.84** | **70.79** | **60.69** |
| sesquiterpenes  **sesquiterpenes** | sesquiterpenes hydrocarbons | α-ylangene | 1373 | 0.15 | 0.14 | 0.23 | 0.02 | 0.23 | 0.01 | 0.26 |
|  |  | α-copaene^S^ | 1378 | 0.06 | 0.10 | 0.04 | 0.01 | 0.01 | 0.01 | 0.09 |
|  |  | β-cubebene | 1391 | 0.06 | - | 0.05 | - | - | - | 0.04 |
|  |  | β-elemene^S^ | 1393 | 0.50 | 0.40 | 0.69 | 1.47 | 1.63 | 0.50 | 1.53 |
|  |  | *trans*-caryophyllene^S^ | 1421 | 1.40 | 2.47 | 3.37 | 3.19 | 3.49 | 3.99 | 2.92 |
|  |  | α-guaiene | 1441 | 0.30 | 0.31 | 0.50 | 0.46 | 0.55 | 0.49 | 0.46 |
|  |  | guaia-3,7-diene | 1446 | 0.07 | 0.12 | 0.17 | 0.01 | 0.17 | 0.01 | 0.18 |
|  |  | α-humulene^S^ | 1456 | 0.67 | 0.74 | 0.54 | 0.38 | 0.56 | 0.46 | 0.66 |
|  |  | alloaromadendrene | 1462 | 0.13 | 0.14 | 0.15 | 0.01 | 0.13 | 0.02 | 0.18 |
|  |  | α-amorphene | 1478 | 0.10 | 0.14 | 0.06 | 0.01 | 0.01 | 0.01 | 0.12 |
|  |  | germacrene D^S^ | 1482 | 0.30 | 0.37 | 0.65 | 0.54 | 0.59 | 0.02 | 0.63 |
|  |  | β-selinene | 1488 | 0.38 | 0.33 | 0.59 | 0.44 | 0.48 | 0.51 | 0.59 |
|  |  | bicyclogermacrene | 1496 | 1.18 | 0.83 | 1.39 | 0.96 | 1.07 | 1.19 | 1.40 |
|  |  | germacrene A | 1505 | 0.71 | 0.58 | 1.43 | 0.93 | 0.94 | 1.25 | 0.87 |
|  |  | γ-cadinene^S^ | 1515 | 0.25 | 0.33 | 0.41 | 0.32 | 0.39 | 0.02 | 0.52 |
|  |  | δ-cadinene | 1525 | 0.44 | 0.66 | 0.70 | 0.54 | 0.53 | 0.58 | 0.81 |
|  |  | *trans*-α-bisabolene | 1545 | 0.32 | 0.25 | 0.42 | 0.01 | 0.31 | 0.02 | 0.20 |
|  | oxygenated sesquiterpenes | berbenone | 1212 | - | 1.49 | - | 0.52 | 0.15 | 1.17 | 0.70 |
|  |  | elemol^S^ | 1553 | 0.13 | 0.09 | 0.12 | 0.02 | 0.12 | 0.01 | 0.15 |
|  |  | nerolidol^S^ | 1568 | 0.19 | 0.06 | 0.11 | 0.01 | 0.07 | 0.02 | 0.01 |
|  |  | caryophyllene oxide^S^ | 1584 | 0.70 | 1.39 | 1.50 | 0.92 | 1.07 | 1.19 | 1.07 |
|  |  | globulol^S^ | 1586 | 0.18 | - | - | - | - | - | - |
|  |  | veridiflorol | 1594 | 4.14 | 1.64 | 0.37 | 0.01 | 0.30 | 0.02 | 1.16 |
|  |  | isospathulenol | 1642 | 0.41 | - | 0.13 | - | 0.19 | - | 0.24 |
|  |  | α-cadinol | 1646 | 0.35 | 0.37 | 0.49 | 0.01 | 0.40 | 0.01 | 0.39 |
|  |  | β-eudesmol^S^ | 1654 | 1.11 | 0.67 | 0.85 | 0.52 | 0.67 | 0.02 | 0.78 |
|  |  | α-eudesmol | 1657 | 1.25 | - | - | - | - | - | - |
|  |  | t-muurolol | 1658 | - | 0.83 | 1.30 | - | 1.06 | 0.01 | 1.06 |
|  | **total sesquiterpenes** |  |  | **15.63** | **14.45** | **16.26** | **11.31** | **15.12** | **11.54** | **17.02** |
| others | [phenylpropane](https://en.wikipedia.org/wiki/Phenylpropene) derivatives | p-allylanisole^S^ | 1199 | 0.08 | - | - | - | - | - | 0.08 |
|  |  | 3-phenylpropenal | 1275 | 0.06 | - | 0.14 | - | 0.06 | - | - |
|  |  | thymol^S^ | 1296 | 0.05 | - | - | - | - | - |  |
|  |  | carvacrol^S^ | 1307 | 0.16 | - | - | - | - | - |  |
|  |  | eugenol^S^ | 1363 | 2.76 | 6.33 | 9.23 | 8.42 | 7.82 | 8.20 | 5.64 |
|  |  | methyleugenol*^S^ | 1409 | 7.15 | 8.52 | 9.71 | 9.21 | 8.20 | 9.33 | 7.95 |
|  |  | *cis*-methylisoeugenol | 1500 | 0.54 | - | 0.62 | - | 0.70 | - | 0.32 |
|  |  | elemicin | 1561 | 0.32 | 0.48 | 0.75 | 0.51 | 0.58 | 0.01 | 0.37 |
|  | other compounds | (Z)-hex-3-en-1-ol^S^ | < 900 | - | - | 0.06 | - | - | - | - |
|  |  | nonan-2-one^S^ | 1094 | - | - | 0.04 | - | - | - | - |
|  |  | decanal^S^ | 1207 | - | - | 0.03 | - | - | - | - |
|  |  | undecan-2-one^S^ | 1295 | - | 0.17 | 0.22 | - | 0.16 | - | 0.12 |
|  |  | pentadecan-2-one^S^ | 1699 | - | - | 0.08 | - | - | - | 0.09 |
|  |  | hexadecanal^S^ | 1818 | - | - | 0.11 | - | - | - | - |
|  | **total others** |  |  | **11.12** | **15.5** | **20.99** | **18.14** | **17.52** | **17.54** | **14.57** |
|  | **total:** |  |  | **92.85** | **92.29** | **92.52** | **98.59** | **94.48** | **99.87** | **92.28** |

^1^Retention index on a MSD model 5975 C and HP-5MS; ^2^HD - hydrodistillation without pretreatment (negative control); ^3^HD-RE - hydrodistillation with reflux extraction pretreatment; hydrodistillation with reflux extraction pretreatment assisted with enzymes: ^4^HD-REX xylanase; ^5^HD-REC cellulase; ^6^HD-REP pectinase; ^7^HD-REPCX pectinase + cellulase + xylanase; ^8^HD-US hydrodistillation with ultrasonic pretreatment; ^9^(-) not detected; * - compounds confirmed by NMR, ^S^ - compounds identified with the standard.

**Table 3.** Chemical composition of rosemary essential oils isolated by hydrodistillation with and without different pretreatments.

|  | **Group** | **Compound** | **RI*^1^*** | **% Total Peak Area** | | | | | | |
| --- | --- | --- | --- | --- | --- | --- | --- | --- | --- | --- |
| **monoterpenes** |  |  |  | HD^2^ | HD-RE^3^ | HD-REX^4^ | HD-REC^5^ | HD-REP^6^ | HD-REPCX^7^ | HD-US^8^ |
|  | monoterpene hydrocarbons | α-pinene^S^ | 941 | 2.25 | 3.28 | 2.93 | 2.77 | 0.93 | 3.36 | 5.88 |
|  |  | camphene^S^ | 956 | 0.47 | 0.62 | 0.54 | 0.50 | 0.19 | 0.59 | 1.19 |
|  |  | verbenene^S^ | 962 | -*^9^* | - | 0.17 | - | - | 0.19 | 0.35 |
|  |  | sabinene^S^ | 979 | - | - | - | - | - | 0.08 | - |
|  |  | β-pinene^S^ | 982 | - | 0.27 | - | - | - | 0.28 | 0.29 |
|  |  | β-myrcene^S^ | 992 | - | - | 0.10 | - | - | - | - |
|  |  | *p*-cymene^S^ | 1030 | 0.01 | 0.42 | 0.42 | 0.01 | 0.02 | 0.15 | 1.03 |
|  |  | δ-car-3-ene^S^ | 1014 | - | 0.33 | 0.33 | - | - | 0.21 | 0.51 |
|  |  | limonene^S^ | 1034 | 0.01 | 0.58 | 0.57 | 0.02 | 0.18 | 0.25 | 1.49 |
|  | oxygenated monoterpenes | 1.8-cineole^S^ | 1038 | 4.34 | 9.49 | 6.19 | 4.55 | 3.44 | 7.44 | 4.24 |
|  |  | linalool*^S^ | 1104 | 5.00 | 5.75 | 5.57 | 4.02 | 3.13 | 5.57 | 1.38 |
|  |  | filifolone | 1105 | - | 0.38 | 0.47 | - | - | 0.29 | 0.29 |
|  |  | α-thujone^S^ | 1110 | - | 0.72 | 0.93 | 0.87 | - | 0.61 | 3.47 |
|  |  | β-thujone^S^ | 1121 | - | 0.38 | 0.53 | 0.38 | - | 0.35 | 1.49 |
|  |  | chrysanthenone | 1129 | 0.20 | 0.72 | 0.99 | 0.45 | - | 0.58 | 0.14 |
|  |  | camphor*^S^ | 1149 | 15.38 | 13.82 | 17.58 | 12.77 | 3.73 | 11.56 | 9.01 |
|  |  | pinocarvone | 1167 | - | 0.73 | 0.28 | 0.68 | - | 0.20 | 0.58 |
|  |  | borneol*^S^ | 1172 | 24.38 | 12.03 | 16.77 | 18.11 | 6.22 | 11.26 | 5.39 |
|  |  | isopinocamphone | 1178 | 0.69 | 1.52 | 1.89 | 1.28 | - | 1.15 | 0.64 |
|  |  | 4-terpineol^S^ | 1182 | 1.00 | 1.70 | 1.31 | 0.90 | 0.97 | 1.24 | 0.52 |
|  |  | *p*-cymen-8-ol^S^ | 1191 | - | - | 0.38 | 0.37 | - | 0.24 | - |
|  |  | α-terpineol^S^ | 1195 | 5.04 | 4.17 | 3.73 | 2.81 | 3.60 | 3.34 | 0.83 |
|  |  | myrtenol^S^ | 1200 | 1.28 | 0.56 | 0.78 | 0.78 | - | 0.53 | - |
|  |  | homomyrtenol | 1208 | 2.39 | - | - | 1.39 | 0.19 | 1.05 | 0.23 |
|  |  | nerol^S^ | 1233 | - | - | - | - | - | 0.27 | - |
|  |  | geraniol^S^ | 1261 | - | - | - | - | - | 0.21 | - |
|  |  | bornyl acetate^S^ | 1287 | - | 0.41 | - | - | 0.33 | 0.23 | 0.47 |
|  |  | α-terpenyl acetate^S^ | 1355 | - | 3.31 | - | - | 3.46 | 2.38 | - |
|  |  | spathulenol | 1581 | - | 0.64 | - | - | 3.81 | 1.28 | - |
|  | **total monoterpenes** |  |  | **62.44** | **61.83** | **62.46** | **52.66** | **30.2** | **52.17** | **39.42** |
| sesquiterpenes | sesquiterpene hydrocarbons | β-elemene^S^ | 1393 | - | - | - | - | 0.39 | 0.22 | - |
|  |  | *trans*-caryophyllene^S^ | 1421 | 1.53 | 2.84 | 2.00 | 0.76 | 1.28 | 1.47 | 0.56 |
|  |  | α-muurolene | 1501 | - | - | 0.22 | - | - | 0.14 | - |
|  |  | α-humulene | 1456 | - | 0.66 | 0.83 | 1.05 | 0.44 | 0.45 | 1.24 |
|  |  | α-guaiene | 1441 | - | - | - | - | - | 0.11 | - |
|  |  | α-copaene | 1378 | - | - | 0.36 | - | - | 0.13 | - |
|  |  | alloaromadendrene | 1462 | - | - | - | - | 0.40 | - | - |
|  |  | β-selinene | 1488 | - | - | - | - | - | 0.13 | - |
|  |  | α-copaene | 1378 | - | - | 0.36 | - | - | 0.13 | - |
|  |  | α-amorphene | 1478 | - | - | 0.40 | - | - | 0.13 | - |
|  |  | γ-cadinene | 1515 | 0.01 | 0.43 | 0.49 | 0.02 | 0.32 | 0.28 | 0.02 |
|  |  | α-calacorene | 1546 | - | - | - | - | - | 0.19 | - |
|  |  | germacrene A | 1505 | - | 0.40 | - | - | - | - | - |
|  |  | δ-cadinene^S^ | 1525 | 0.81 | 1.22 | 1.31 | 0.50 | 0.83 | 0.60 | 0.69 |
|  | oxygenated sesquiterpenes | berbenone* | 1212 | 21.76 | 12.17 | 14.40 | 13.24 | 5.55 | 9.93 | 5.56 |
|  |  | spathulenol | 1581 | - | 0.64 | - | - | 3.81 | 1.28 | - |
|  |  | caryophyllene oxide | 1584 | - | 0.67 | - | - | 1.88 | 0.92 | - |
|  |  | veridiflorol | 1594 | - | - | 1.14 | 8.01 | 11.95 | 1.85 | 14.32 |
|  |  | α-caryophylladienol | 1640 | - | - | - | 0.43 | 2.60 | 0.83 | 1.11 |
|  |  | α-cadinol | 1646 | - | 0.63 | 0.47 | - | 1.21 | 0.82 | - |
|  |  | β-eudesmol | 1654 | - | 0.84 | - | - | 3.80 | 1.15 | - |
|  |  | t-muurolol | 1658 | - | 1.54 | 0.19 | 1.76 | 6.31 | 1.84 | 0.13 |
|  |  | manool | 2055 | - | 0.45 | - | 13.47 | 13.65 | 1.71 | 22.05 |
|  | **total sesquiterpenes** |  |  | **24.11** | **23.53** | **21.81** | **39.24** | **56.43** | **25.1** | **45.68** |
| others | phenylpropane derivatives | thymol^S^ | 1296 | - | - | 0.22 | - | - | 0.20 | - |
|  |  | carvacrol^S^ | 1307 | - | - | - | - | - | 0.32 | - |
|  |  | eugenol^S^ | 1363 | - | 5.70 | - | - | 3.44 | 4.24 | - |
|  |  | methyleugenol^S^ | 1409 | 0.01 | 3.56 | 0.17 | 0.02 | 2.65 | 2.82 | - |
|  |  | elemicin | 1561 | - | 0.46 | - | - | 0.46 | 0.39 | - |
|  | other compounds | oct-1-en-3-ol | 983 | - | - | 0.24 | - | - | - | - |
|  |  | methyl jasmonate^S^ | 1651 | - | - | 0.30 | - | - | 0.62 | - |
|  |  | hexadecan-1-ol^S^ | 1885 | - | - | - | - | 0.23 | 0.13 | - |
|  | **total others** |  |  | **0.01** | **9.72** | **0.93** | **0.02** | **6.78** | **8.72** | **0** |
|  | **total:** |  |  | **86.56** | **95.07** | **85.2** | **91.92** | **93.41** | **85.99** | **85.1** |

^1^Retention index on a MSD model 5975 C and HP-5MS; ^2^HD - hydrodistillation without pretreatment (negative control); ^3^HD-RE - hydrodistillation with reflux extraction pretreatment; hydrodistillation with reflux extraction pretreatment assisted with enzymes: ^4^HD-REX xylanase; ^5^HD-REC cellulase; ^6^HD-REP pectinase; ^7^HD-REPCX pectinase + cellulase + xylanase; ^8^HD-US hydrodistillation with ultrasonic pretreatment; ^9^(-) not detected; * - compounds confirmed by NMR*,* ^S^ - compounds identified with the standard.
